# Gut commensal *Phascolarctobacterium faecium* retunes innate immunity to mitigate obesity and metabolic disease in mice

**DOI:** 10.1038/s41564-025-01989-7

**Published:** 2025-05-06

**Authors:** Rebeca Liébana-García, Inmaculada López-Almela, Marta Olivares, Marina Romaní-Pérez, Paolo Manghi, Alba Torres-Mayo, Verónica Tolosa-Enguís, Alejandra Flor-Duro, Clara Bullich-Vilarrubias, Teresa Rubio, Valerio Rossini, Nicola Segata, Yolanda Sanz

**Affiliations:** 1 Microbiome Innovation in Nutrition and Health Research Unit, Institute of Agrochemistry and Food Technology, Spanish National Research Council (IATA-CSIC), Valencia, Spain; 2 Department CIBIO, University of Trento, Trento, Italy; 3 Research and Innovation Center, Edmund Mach Foundation, San Michele all’Adige, Italy

**Keywords:** Microbial communities, Metabolic disorders

## Abstract

The gut microbiota may protect against obesity and chronic metabolic conditions by regulating the immune response to dietary triggers. Yet the specific bacteria that control the overactivation of the immune system in obesity and their mode of action remain largely unknown. Here we surveyed 7,569 human metagenomes and observed an association between the gut symbiont *Phascolarctobacterium faecium* and non-obese adults regardless of nationality, sex or age. In a mouse model of diet-induced obesity, we confirmed the specificity of *P. faecium* DSM 32890 anti-obesogenic properties compared with other species of the same genus. *P. faecium* reversed the inflammatory phenotype associated with obesity. Specifically, *P. faecium* promoted polarization of alternatively activated macrophages (M2), which reversed the obesity-induced increase in gut-resident type 1 innate lymphoid cells. This resulted in mitigation of glucose intolerance, adiposity and body weight gain irrespective of treatment with live or pasteurized bacteria. The metabolic benefits were independent of the adaptive immune system, but they were abolished by an inhibitor of M2 polarization in mice. *P. faecium* directly promoted M2-macrophage polarization through TLR2 signalling and these effects seemed to be independent of gut microbiota changes. Overall, we identify a previously undescribed gut commensal bacterium that could help mitigate obesity and metabolic comorbidities by retuning the innate immune response to hypercaloric diets.

## Abstract (web summary)

Gut microbiome member, *Phascolarctobacterium faecium*, mitigates the inflammatory phenotype associated with obesity by retuning the innate immune response in a mouse model of diet-induced obesity.

## Main

Obesity is a complex disease with an increasing worldwide prevalence mainly due to the adoption of a Westernized lifestyle. Obesity contributes to the burden of non-communicable diseases and causes substantial deterioration in quality of life and well-being, placing a strain on healthcare systems, economic productivity and social resources^1^. Continual exposure to dietary-related inflammatory insults, such as refined sugars and saturated fats, profoundly impacts on the gut microbiota and impairs gut barrier function and intestinal immunity^2,3^. Diet-related changes in these systems secondarily influence adiposity, insulin resistance and other obesity hallmarks. Indeed, growing evidence indicates that a skewed gut immunity towards an inflammatory state is an early trigger of obesity and its comorbidities including type 2 diabetes and fatty liver disease^4^.

The enhanced recruitment of pro-inflammatory macrophages evidenced in the stomach, duodenum and colon of people with obesity correlates with body weight and unhealthy lifestyle behaviours such as diets low in vegetables or high in processed meats^5^. Similarly, increases in other leucocyte populations in the intestinal mucosa, including type 1 innate lymphoid cells (ILC1s) and pro-inflammatory T helper (Th)1 cells, have been reported in obesity^6,7^. The gut inflammatory response to energy-dense diets is thought to prime downstream inflammation in metabolic organs including visceral adipose tissue, a principal driver of obesity-related insulin resistance^8,9^. As a matter of fact obese mice treated with the anti-inflammatory drug 5-aminosalicylic acid showed improvements in glucose tolerance and insulin sensitivity^10^, confirming the contribution of gut inflammation to obesity complications. Regarding the underlaying mechanisms, it has been hypothesized that obesogenic diets act as pro-inflammatory signals that disrupt the communication between immune cells and intestinal epithelial cells (IECs), resulting in metabolic derangements^11^. Diet–immune interactions may also affect the gut microbiota and, thereby, drive metabolic dysfunction^12^. We and others found that obesogenic diets diminish the production of intestinal interleukin (IL)-22 (refs. ^13,14^), which normally induces antimicrobial peptide (AMP) secretion by IECs and immunoglobulin A (IgA) production, thereby regulating gut microbiota composition^13^. In mice, supplementation of an obesogenic diet with inulin restored IL-22 production in a microbiota-dependent manner, mitigating the main effects of obesity^13^.

Epidemiological studies indicate that obesity and obesity risk are associated with gut microbiota alterations, which could be restored through interventions, constituting a promising tool for nutrition and medicine^15,16^. Along this line, recent years have seen substantial efforts aimed at testing new isolated intestinal bacteria for obesity prevention in preclinical models (reviewed in ref. ^16^). For example, a strain of *Holdemanella biformi*s improved glucose homeostasis in diet-induced obesity (DIO) models through modulation of the enteroendocrine system^17^. Similarly, *Akkermansia muciniphila* administration mitigated obesity-associated alterations by protecting the gut barrier via mucus production, Toll-like-receptor (TLR)2 signalling^18^ and increased glucagon-like peptide 1 (GLP-1) secretion^19^. In this context, we aimed to elucidate the potential of a previously undescribed gut bacterial species, *Phascolarctobacterium faecium*, to mitigate obesity, for which there is no direct evidence today. *P. faecium*, a Gram-negative, non-motile, non-sporulating, anaerobic bacterium belonging to the phylum Firmicutes, is a frequent colonizer of the human gut^20^. In particular, our study was motivated by previous negative associations observed between this bacterium species and the risk of developing excessive weight gain in children^15^, as well as the percent of body fat and fasting insulin in adults with obesity^21^. Besides, *Phascolarctobacterium* has been tied to greater weight loss under a lifestyle intervention in overweight adults^22^. Here we robustly linked *P. faecium* to a non-obese phenotype, supporting its relevance as a biomarker in a large meta-analysis in humans. We also evidenced the anti-obesogenic potential of the strain *P. faecium* DSM 32890 in a murine model of DIO. Furthermore, we combined multiple loss-of-function in vivo and in vitro study models to identify the mode of action of the bacterium over the gut immune cell circuity, ultimately reducing adiposity and glucose intolerance.

## Results

### Large human multicohort study links *P. faecium* to normal weight

**Fig. 1:**
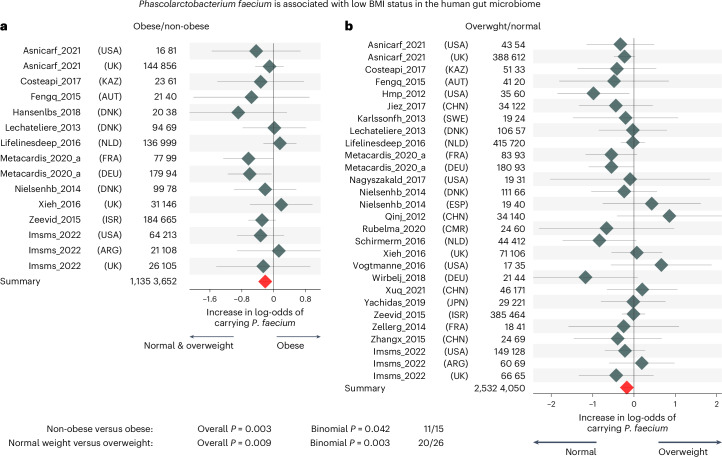
The species *P. faecium* is linked with lower BMI in two large pooled analyses of the human gut microbiome. **a**, Forest plot of a random-effect meta-analysis of the presence of *P. faecium* (3,652 non-obese versus 1,135 obese participants). **b**, Forest plot of a random-effect meta-analysis of the presence of *P. faecium* (4,050 normal-weighted and 2,532 overweight participants). Study name, sample sizes and nationalities are reported. The overall logistic regression meta-analysis significance was assessed using two-tailed standard *t*-test against the null hypothesis of a zero effect size. Binomial tests were used to assess the overproportion of single-dataset tests leaning towards one side of the plot assuming an expected proportion of 50%. Values are presented as mean ± 95% confidence intervals.

**Extended Data Fig. 1:**
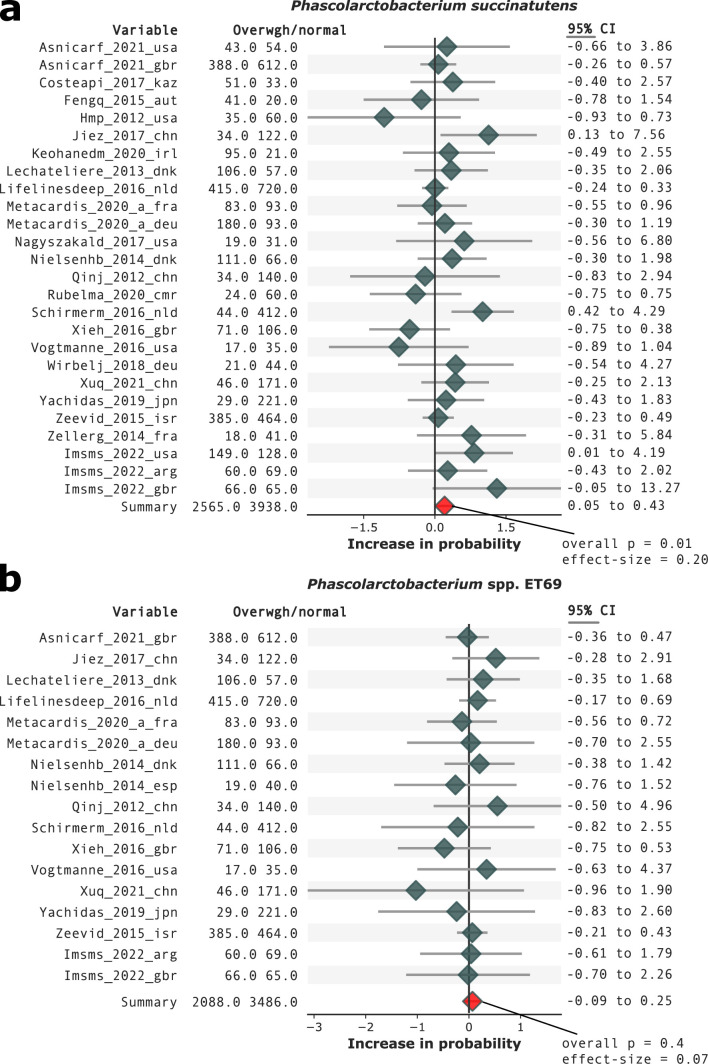
Meta-analysis of association of *Phascolarctobacterium* species with BMI in a large-scale human cohort. (**a**) Forest plot of a random effect meta-analysis of the presence of the species *P. succinatutens* (3,938 normal-weighted participants and 2,565 overweight). (**b**) Forest plot of a random effect meta-analysis of the presence of the species of *Phascolarctobacterium* spp. ET69 (3,486 normal-weighted participants and 2,088 overweight). Study name, sample sizes, and nationalities are reported. The overall logistic regression meta-analysis significance was assessed through two-tailed standard t-test against the null-hypothesis of a zero effect-size. Binomial tests assess the overproportion of single-datasets tests leaning toward one side of the plot assuming an expected proportion of 50%. Values are presented as mean ± 95% confidence intervals.

We first assessed whether the presence of *P. faecium* was associated with body weight in a large collection of metagenomic studies. We collected 7,569 human metagenomes from healthy adult individuals with metadata available in curatedMetagenomicData 3 (ref. ^23^), that were taxonomically profiled using MetaPhlAn 4. We then created two datasets: the first encompassed 4,050 individuals with a body mass index (BMI) < 25 (normal weight) and 2,532 individuals with BMI ≥ 25 (overweight) (15 nationalities, 28 studies); the second totalled 3,652 individuals with BMI < 30 (non-obese) and 1,135 individuals with BMI ≥ 30 (obese) (8 nationalities, 15 studies). The overall prevalence of *P. faecium* was 41% and 33% in normal and overweight individuals, respectively, and 34% and 28% in non-obese and obese individuals. Two multicohort meta-analyses in each dataset revealed that *P. faecium* was 16% and 19% less likely to be detected in overweight (*P* = 0.003) and obese (*P* = 0.009) participants, respectively (Fig. 1a,b, and Supplementary Tables 1 and 2). Negative associations in both analyses were also higher than expected by chance (binomial *P* = 0.003 and 0.040). These results remained significant when sex or age was added as covariates (*P* = 0.008 and 0.009, and 0.008 and 0.014 in the two datasets). Two additional pooled analyses on another two *Phascolarctobacterium* spp. (*P*. spp. ET69 and *P. succinatutens*) identified in the datasets revealed a 23% greater presence of *P. succinatutens* in overweight individuals (*P* = 0.009) (Extended Data Fig. 1a) and a lack of correlation of *P*. spp. ET69 with BMI classes (*P* = 0.4) (Extended Data Fig. 1b). Therefore, only *P. faecium* associates with a non-obese phenotype in the adult human population regardless of nationality, sex or age.

### *P. faecium* curbs adiposity and restores glucose homeostasis

**Fig. 2:**
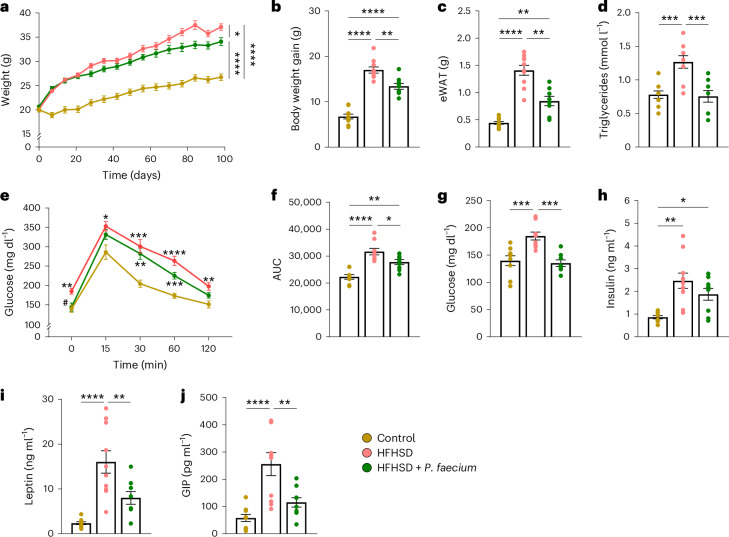
*P. faecium* DSM 32890 curbs body weight gain and adiposity, and restores glucose homeostasis in diet-induced obesity. **a**–**f**, Body weight evolution (**a**), body weight gain (**b**), weight of eWAT (**c**), plasma triglyceride levels (**d**), blood glucose levels after an oral glucose load (2 g kg^−1^) (**e**) and area under the curve (AUC) at week 10 of intervention (**f**). **g**–**j**, Fasting glucose (**g**), insulin (**h**), leptin (**i**) and GIP (**j**) levels in plasma. Control and HFHSD *n* = 10, HFHSD + *P. faecium* *n* = 9. Values are presented as mean ± s.e.m. of *n* biological replicates shown as individual dots. Significant differences were assessed using one- or two-way ANOVA followed by a post hoc Tukey’s test; *P < 0.05. In **e**, ‘*’ is used when compared to control diet and ‘^#^’ when compared to HFHSD. *P < 0.05, **P < 0.01, ***P < 0.001, ****P < 0.0001 and ^#^*P* < 0.05. Source data

**Extended Data Fig. 2:**
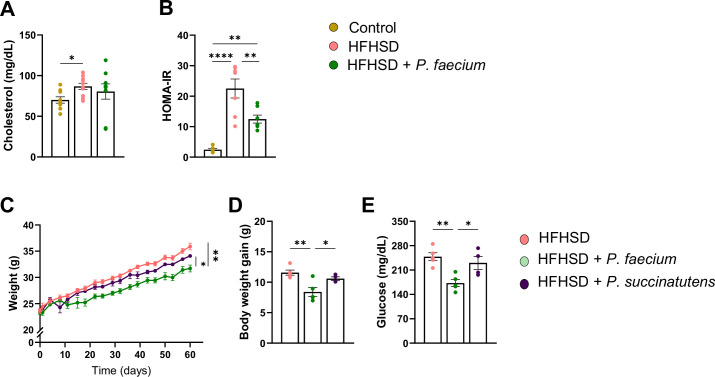
*P. faecium* mitigates the metabolic disturbances triggered by an obesogenic diet. (**a**) Cholesterol concentration in plasma (mg/dL) and (**b**) the homeostatic model assessment for insulin resistance (HOMA-IR) index, (**c**) Body weight evolution, (**d**) body weight gain (g) (**e**) Fasting glucose (mg/dL). Values presented are mean ± s.e.m. Control and HFHSD n = 10, HFHSD + *P. faecium* n = 9 (A and B); HFHSD and HFHSD + *P. faecium* n = 5, HFHSD + *P. succinatutens* n = 4 (**c–e**). Data points show measurements from each mouse sample. Significant differences were assessed by one- or two-way ANOVA followed by a post-hoc Tukey test. Asterisk (*) represent statistical significance at *p < 0.05, **p < 0.01, and **** p < 0.0001. Source data

Given our findings in humans, we next evaluated the effects of the DSM 32890 strain of *P. faecium* in obese mice to confirm its potential causal role. This *P. faecium* strain was isolated from the faeces of a metabolically healthy subject and, therefore, evaluated for its possible contribution to protecting the metabolic phenotype. Wild-type (WT) mice exhibited greater body weight and epididymal white adipose tissue (eWAT) gains on the high-fat high-sugar diet (HFHSD) than on the control diet (CD), while *P. faecium* treatment limited increases in both anthropometric features in HFHSD-fed mice (Fig. 2a–c). *P. faecium* also normalized the plasma levels of triglycerides in HFHSD-fed mice without affecting cholesterol levels (Fig. 2d and Extended Data Fig. 2a). Similarly, *P. faecium* improved whole-body glucose clearance, restored basal glucose levels and the homeostatic model assessment of insulin resistance (HOMA-IR) index (Fig. 2e–g and Extended Data Fig. 2b), but not insulinemia (Fig. 2h). Moreover, *P. faecium* treatment attenuated the HFHSD-induced gains in circulating leptin and glucose-dependent insulinotropic polypeptide (GIP) (Fig. 2i,j). Finally, to confirm the specificity of the *P. faecium* effects, we performed a comparative study between *P. faecium* and *P. succinatutens* DSM 22533 administered to the same DIO mouse model. Unlike *P. faecium*, the administration of *P. succinatutens* did not prevent body weight gain or reduce glucose blood levels in obese mice (Extended Data Fig. 2c–e). Overall, these results demonstrate the uniqueness of *P. faecium* in mitigating the metabolic disturbances triggered by an obesogenic diet.

### *P. faecium* reduces obesity-induced inflammation

**Fig. 3:**
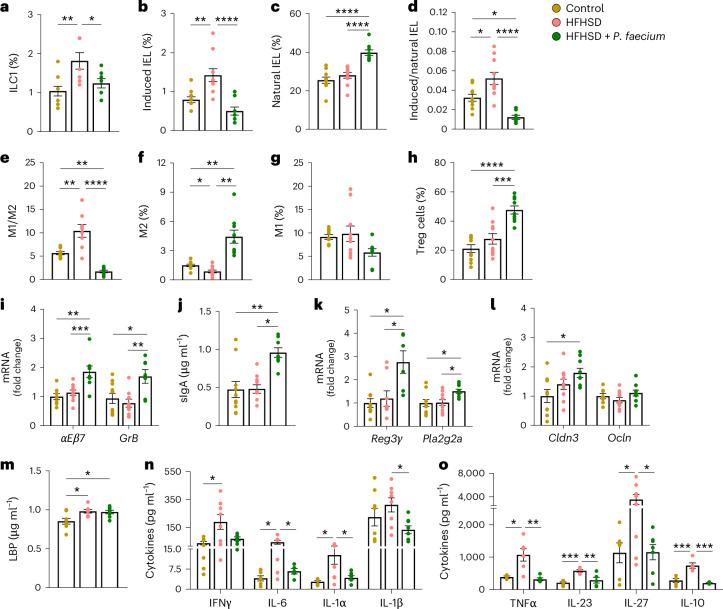
*P. faecium* DSM 32890 reduces HFHSD-induced intestinal and systemic inflammation. **a**–**d**, Analysis of the small intestine: ILC1s (percentage of T-bet^+^ IFNγ^+^ cells from LIN^−^ cells) in total intestinal epithelial cells (**a**); induced IELs (percentage of CD3^+^CD2^+^CD5^+^ TCRαβ^+^ cells from CD45^+^ cells) in the total intestinal epithelial cells (**b**); natural IELs (percentage of CD3^+^CD2^−^CD5^−^ TCRγδ^+^ cells from CD45^+^ cells) in the total intestinal epithelial cells (**c**) and induced and natural IEL ratio (**d**). **e**–**g**, Ratio of M1 to M2 macrophages (**e**), alternative activated (M2) macrophages (percentage of CD206^+^ Arg1^+^ cells from F4/80^+^ cells) (**f**) and pro-inflammatory (M1) macrophages (percentage of CD80^+^ iNOS^+^ cells from F4/80^+^ cells) (**g**) in the total lamina propria cells. **h**, Treg cells (percentage of CD25^+^ Foxp3^+^ cells from CD4^+^ cells) in the total lamina propria cells. **i**, mRNA relative expression of *αEβ7* integrin and granzyme B (*GrB*) in the small intestine. **j**, Secretory immunoglobulin A (sIgA) level in the caecal content. **k**,**l**, mRNA relative expression of *Reg3γ* and *Pla2g2a* (**k**) and of claudin (*Cldn)* 3 and occludin (*Ocln*) (**l**) in the small intestine. **m**, Plasma LBP levels. **n**,**o**, Plasma levels of indicated cytokines. Control and HFHSD *n* = 10, and HFHSD + *P. faecium* *n* = 9 for **a**–**l**; control, HFHSD and HFHSD + *P. faecium* *n* = 8 for **m**–**o**. Values are presented as mean ± s.e.m. of *n* biological replicates shown as individual dots. Significant differences were assessed using one-way ANOVA or Kruskal–Wallis test followed by the corresponding post hoc test; *P < 0.05, **P < 0.01, ***P < 0.001 and ****P < 0.0001. Source data

Compared with the CD, mice on HFHSD showed increased intestinal ILC1s and induced intra-epithelial lymphocyte (IEL) proportions, which were normalized by *P. faecium* treatment (Fig. 3a,b). Moreover, *P. faecium* increased the percentage of natural IELs and attenuated the induced/natural IEL ratio, enhanced by HFHSD (Fig. 3c,d). The HFHSD also triggered a shift in intestinal macrophages, increasing the dominance of those with a pro-inflammatory phenotype (M1) compared with alternatively activated macrophages (M2), as shown by the higher M1/M2 macrophage ratio (Fig. 3e). In contrast, *P. faecium* treatment reduced the M1/M2 ratio by boosting the M2 population and decreasing the M1 population (*P* = 0.052) (Fig. 3e–g). Finally, *P. faecium* treatment substantially increased the number of gut T regulatory (Treg) cells as compared with the CD and HFHSD-fed mice (Fig. 3h).

We next analysed the gut expression of genes related to lymphocyte anchoring (*αEβ7* integrin, also known as CD103) and cytolytic activity (granzyme B (*GrB*)), finding that *P. faecium* treatment significantly increased the expression of both in HFHSD-fed mice (Fig. 3i). The same response was observed for secretory IgA (sIgA) and the AMPs *Pla2g2*a and *Reg3*γ (Fig. 3j,k). In contrast, *P. faecium* treatment did not affect the expression of the gut barrier markers (Fig. 3l) or plasma levels of lipopolysaccharide-binding protein (LBP) (Fig. 3m). HFHSD triggered an increase in most of the plasma cytokines quantified (7 of 8), whereas *P. faecium* normalized their levels to those of CD-fed mice (Fig. 3n,o). These results suggest that *P. faecium* buffers the HFHSD-induced dysregulation of intestinal immune cells and improves gut defence mechanisms, thereby reducing systemic inflammation.

### *P. faecium* protects metabolic health regardless of adaptive immunity or viability

**Fig. 4:**
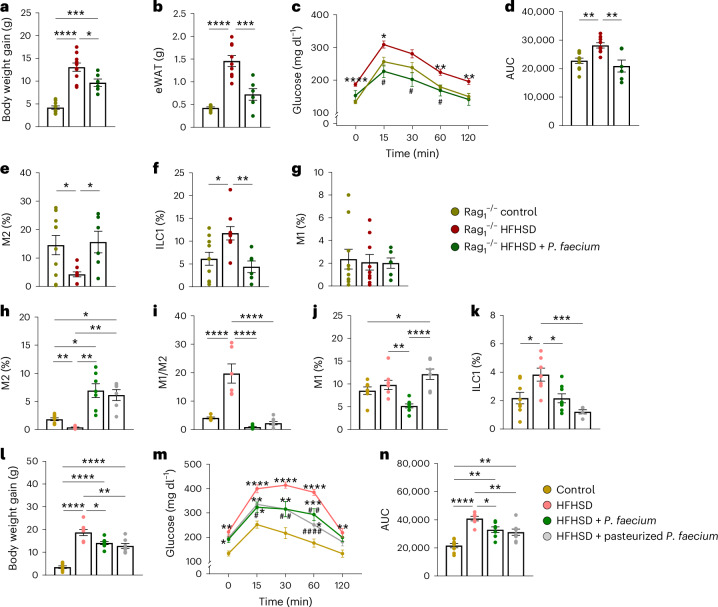
*P. faecium* DSM 32890 protects metabolic health in obesity in the absence of adaptive immunity and irrespective of its viability in vivo*.* **a**–**d**, Body weight gain (**a**) and weight of eWAT (**b**), blood glucose levels after an oral load of glucose (2 g kg^−1^) (**c**) and AUC (**d**). **e**–**g**, Alternative activated (M2) macrophages (percentage of CD206^+^ Arg1^+^ cells from F4/80^+^ cells) in the total lamina propria cells (**e**), ILC1s (percentage of T-bet^+^ IFN-γ^+^ cells from LIN^−^ cells) in total intestinal epithelial cells (**f**) and pro-inflammatory (M1) macrophages (percentage of CD80^+^iNOS^+^ cells from F4/80^+^ cells) in the total lamina propria cells (**g**). **h**–**k**, Analysis of the small intestine: alternative activated (M2) macrophages (percentage of CD206^+^ Arg1^+^ cells from F4/80^+^ cells) in the total lamina propria cells (**h**), ratio of M1 to M2 (**i**), pro-inflammatory (M1) macrophages (percentage of CD80^+^ iNOS^+^ cells from F4/80^+^ cells) in the total lamina propria cells (**j**) and ILC1s (percentage of T-bet^+^ IFN-γ^+^ cells from LIN^−^ cells) in total intestinal epithelial cells (**k**). **l**–**n**, Body weight gain (**l**), blood glucose levels after an oral load of glucose (2 g kg^−1^) (**m**) and AUC at week 10 of intervention (**n**). *Rag1*^−*/*−^ control and HFHSD *n* = 10, and *Rag1*^−*/*−^ HFHSD + *P. faecium* *n* = 6 for **a**–**g**; WT mice *n* = 8 for **h**–**n**. Values are presented as mean ± s.e.m. of *n* biological replicates shown as individual dots. Significant differences were assessed using one- or two-way ANOVA followed by a post hoc Tukey’s test; *P < 0.05. In **c** and **m**, ‘*’ is used when compared to the control diet and ‘^#^’ when compared to HFHSD. *P < 0.05, **P < 0.01, ***P < 0.001, ****P < 0.0001, ^#^*P* < 0.05, ^##^*P* < 0.01 and ^####^*P* < 0.0001. Source data

To test the role of adaptive immunity in the metabolic effects of *P. faecium*, we utilized *Rag1*^−/−^ mice lacking mature B and T cells. *Rag1*^−/−^ mice gained weight and fat mass under HFHSD feeding accompanied by glucose intolerance (Fig. 4a–d). *P. faecium* treatment prevented these metabolic hallmarks despite the absence of adaptive immunity (Fig. 4a–d). Similar to WT mice, *P. faecium* boosted the proportion of M2 macrophages in *Rag1*^−/−^ mice and prevented the HFHSD-induced increase in ILC1s (Fig. 4e,f), whereas M1 macrophages were unaffected by the diet or the bacterium (Fig. 4g).

We next questioned whether the immunomodulatory effects of *P. faecium* on M2 macrophages depended on its viability by treating HFHSD-fed mice with live or pasteurized (non-viable) bacteria. Results showed that both non-viable and viable *P. faecium* also triggered the intestinal M2 activation (Fig. 4h), equally reducing the obesity-induced M1/M2 ratio increase (Fig. 4i), although differences were observed in the abundance of M1 macrophages (Fig. 4j). Similarly, both treatments equally reduced the percentage of ILC1s (Fig. 4k). Finally, pasteurized *P. faecium* exerted the same metabolic benefits as live bacteria in terms of body weight gain reduction and oral glucose tolerance (Fig. 4l–n). These results show that the anti-obesogenic effects of *P. faecium* are not mediated by adaptive immunity, and that the M2-mediated immune effects of *P. faecium* do not depend on its viability.

### *P. faecium* exerts metabolic benefits by polarizing M2 macrophages

**Fig. 5:**
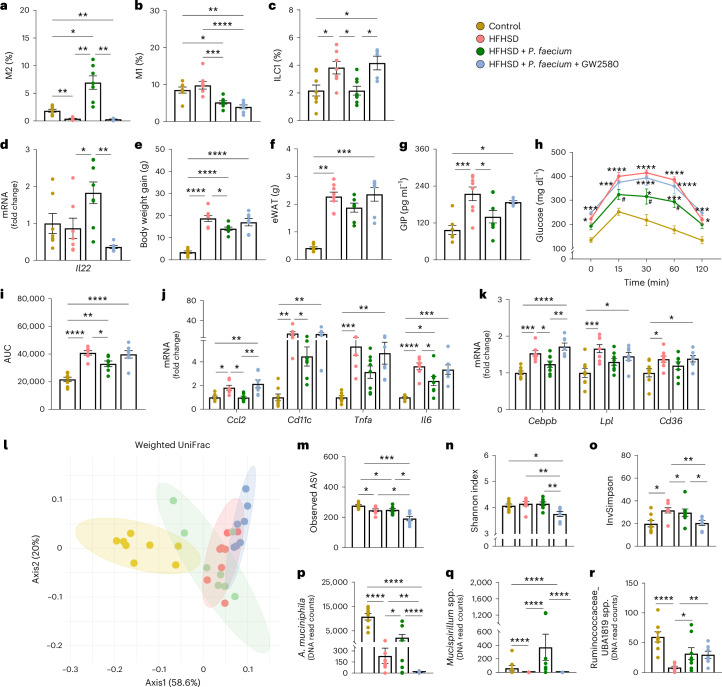
*P. faecium* DSM 32890 exerts metabolic benefits through M2-macrophage polarization. **a**,**b**, Analysis of the small intestine: alternative activated (M2) macrophages (percentage of CD206^+^ Arg1^+^ cells from F4/80^+^ cells) (**a**) and pro-inflammatory (M1) macrophages (percentage of CD80^+^ iNOS^+^ cells from F4/80^+^ cells) (**b**) in the total lamina propria cells. **c**,**d**, ILC1s (percentage of T-bet^+^ IFN-γ^+^ cells from LIN^−^ cells) in total intestinal epithelial cells (**c**) and mRNA relative expression of IL-22 (**d**). **e**–**i**, Body weight gain (**e**) and weight of eWAT (**f**), fasting GIP levels in plasma (**g**), blood glucose levels after an oral load of glucose (2 g kg^−1^) (**h**) and AUC (**i**). **j**,**k**, mRNA relative expression of immune makers (**j**) and lipid metabolic (**k**) genes in the eWAT. **l**–**o**, Analysis of the microbiota in the caecal content: beta-diversity based on weighted UniFrac distances (**l**), observed ASVs (**m**), Shannon diversity index (**n**) and Inverse Simpson index (**o**). **p**–**r**, Normalized abundance of *Akkermansia muciniphila* (**p**), *Mucispirillum* spp. (**q**) and Ruminococcaceae_UBA1819 spp*.* (**r**). Control, HFHSD and HFHSD + *P. faecium* *n* = 8 and HFHSD + *P. faecium* + GW2580 *n* = 7. Values are presented as mean ± s.e.m. of *n* biological replicates shown as individual dots. Significant differences were assessed using one- or two-way ANOVA or Kruskal–Wallis test followed by the corresponding post hoc test. Non-parametric methods were applied for statistical analysis of alpha diversity and differential abundance analysis was performed using the DESeq2 v.1.36 R package. The resulting *P* values were corrected using the Benjamini–Hochberg (BH) FDR procedure; *P < 0.05. In **h**, ‘*’ is used when compared to the control diet and ‘^#^’ when compared to HFHSD. *P < 0.05, **P < 0.01, ***P < 0.001, ****P < 0.0001 and ^#^*P* < 0.05. Source data

**Extended Data Fig. 3:**
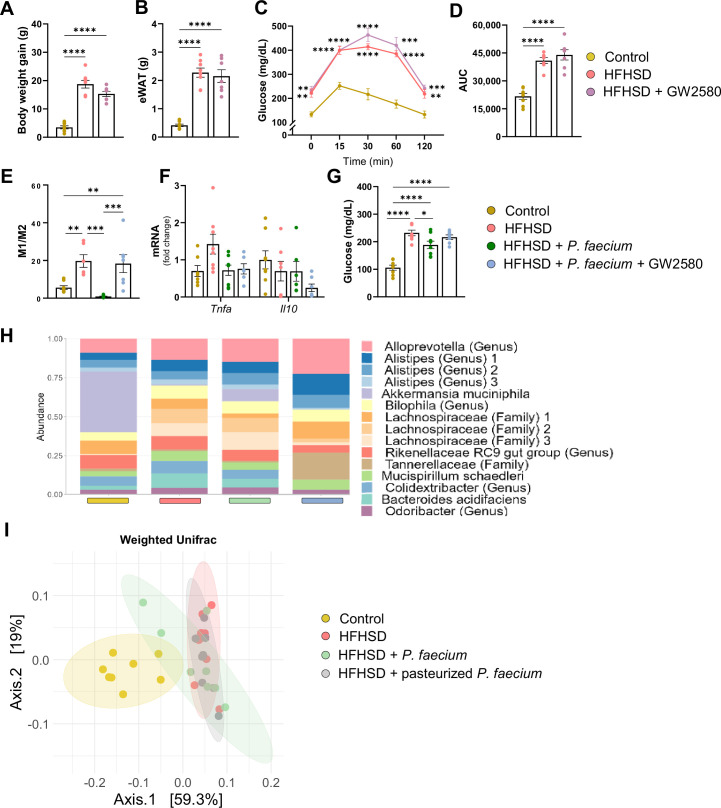
*P. faecium* exerts metabolic benefits by polarizing M2 macrophages. (**a**) Body weight gain (g), (**b**) weight (g) of epididymal white adipose tissue (eWAT), (**c**) blood glucose levels after an oral load of glucose (2 g/kg) and (**d**) area under the curve (AUC) at week 10 of intervention. (**e**) ratio of M1 to M2 macrophages (**f**) mRNA relative expression of indicated genes in the small intestine, (**g**) fasting glycemia (mg/dL), (**h**) mean relative abundance by group of the 15 most abundant genera/families, (**i**) Beta-diversity based on weighted UniFrac distances. Values are presented as mean (Control, HFHSD and HFHSD + *P. faecium* n = 8; HFHSD + *P. faecium* + GW2580 and HFHSD + GW2580 n = 7) ± s.e.m. Data points show measurement s from each individual mouse sample. Significant differences were assessed by one- or two-way ANOVA. Differential abundance analysis was performed using the DESeq2 v.1.36R package. Asterisk (*) represent statistical significance at *p < 0.05, **p < 0.01, ***p < 0.001 and ****p < 0.0001. Source data

We tested whether the induction of M2 macrophages is the mechanism through which *P. faecium* exerts metabolic benefits in obesity. To this end, we co-administered GW2580, which specifically inhibits M2 polarization as described elsewhere^24^, to HFHSD-fed mice. GW2580 administration alone did not affect body weight gain, adiposity or glucose tolerance in HFHSD-fed mice (Extended Data Fig. 3a–d). We confirmed that GW2580 co-administration blocked the *P. faecium-*induced increase in intestinal M2 macrophages (Fig. 5a) and the reduction of the M1/M2 ratio (Extended Data Fig. 3e), without affecting M1 macrophages (Fig. 5b). Remarkably, inhibition of M2-macrophage polarization also tempered some of the beneficial effects of *P. faecium*, including the reduction in ILC1 levels (Fig. 5c) and the increase in *Il22* expression in the small intestine (Fig. 5d). However, it did not affect *Tfna* or *Il10* expression (Extended Data Fig. 3f). Of note, the beneficial effects of *P. faecium* on weight gain, adipose tissue expansion and GIP levels were lost when M2-macrophage polarization was inhibited (Fig. 5e–g). Similarly, the positive effects on glucose tolerance and basal glycaemia elicited by *P. faecium* treatment were partly lost in mice co-treated with GW2580 (Fig. 5h,i and Extended Data Fig. 3g). In the eWAT, the M2 blockade with GW2580 impeded the *P. faecium-*induced decrease in *Ccl2* expression (coding for the monocyte chemoattractant protein 1), which is involved in immune cell recruitment in WAT (Fig. 5j). Similarly, GW2580 co-administration lessened the *P. faecium*-induced decrease in *Cd11c*, *Tnfa* and *Il6* expression in this tissue (Fig. 5j). The changes in the inflammatory milieu also impacted the metabolic functioning of the WAT. Inhibition of M2 polarization reverted the *P. faecium-*stimulated reduction in adipogenesis, as reflected by *Cebpb* expression (Fig. 5k). These findings indicate that M2 macrophages are key mediators of the metabolic benefits of *P. faecium*.

### *P. faecium* restores HFHSD-altered microbiota, requiring viability and M2 polarization

The analysis of the microbiota structure in mice revealed that *P. faecium* partly normalized the HFHSD-induced changes, an effect that was not observed when *P. faecium* was co-administered with the M2 inhibitor (Fig. 5l and Extended Data Fig. 3h) or pasteurized (Extended Data Fig. 3i). All HFHSD-fed groups had a lower microbial richness than the mice fed a CD (Fig. 5m). In addition, *P. faecium* plus GW2580 showed a reduced Shannon’s alpha-diversity index compared with the control, and a normalized population evenness (Fig. 5n,o). Regarding taxonomy, 96 amplicon sequence variants (ASVs) identified at genus/group or species levels were modified by any of the treatments compared with the control (Supplementary Table 3). Specifically, 9 of the 96 ASVs were modified by *P. faecium* treatment compared with non-treated HFHSD-fed mice. *P. faecium* treatment reversed the HFHSD-induced reduction of two mucus-dwelling bacteria, *A. muciniphila* and *Mucispirillum* spp., and these effects were lost when the M2 inhibitor was co-administered (Fig. 5p,q). A similar finding was observed for the butyrate producer Ruminococcaceae_UBA1819 spp., but this was not blunted by GW2580 (Fig. 5r). *P. faecium* treatment thus prevented the loss of functionally relevant bacterial taxa caused by the HFHSD, and M2-macrophage blockade tempered some of the benefits of *P. faecium* in the intestinal ecosystem.

### *P. faecium* promotes M2-macrophage polarization via TLR2

**Fig. 6:**
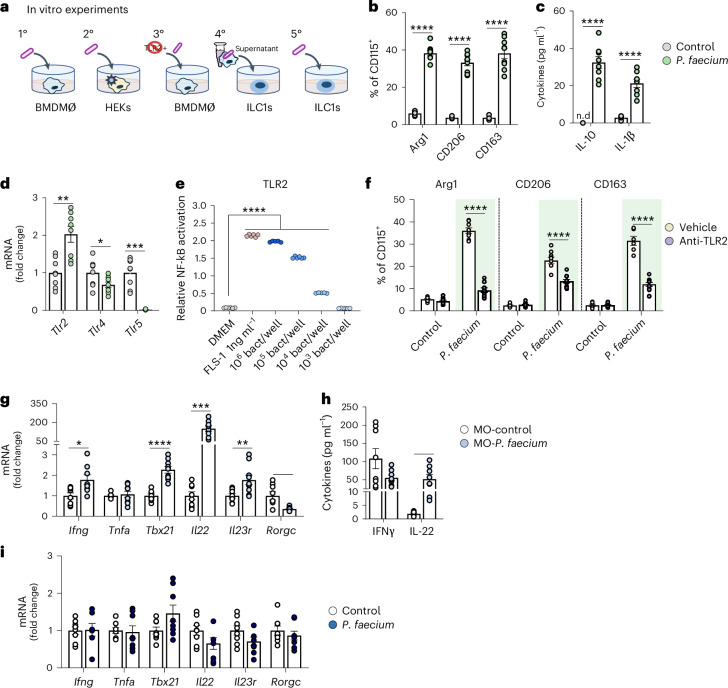
*P. faecium* DSM 32890 directly promotes M2-macrophage polarization via TLR2. **a**, Schema of in vitro experiments. **b**, Arg1, CD206 and CD163 percentages of MHC-II^+^CD11c^+^CD11b^high^ CD115^+^ in BMDMØ cell cultures (*n* = 9). **c**, Cytokine levels in the BMDMØ culture supernatants (*n* = 8). **d**, mRNA relative expression of TLRs in BMDMØ cultures exposed to PBS (grey) or *P. faecium* (green) for 6 h (*n* = 8). **e**, Relative NF-kB activation in response to different *P. faecium* concentrations in HEK293-Blue-hTLR2 cells (*n* = 6). **f**, Arg1, CD206 and CD163 percentages of MHC-II^+^CD11c^+^CD11b^high^ CD115^+^ in BMDMØ cell cultures co-exposed to *P. faecium* (1:10 cell/bacteria ratio) and/or anti-TLR2 (1 μg ml^−1^) (*n* = 12). **g**, mRNA relative expression of genes in intestinal ILC1 cultures exposed to control BMDMØ supernatants (MO-control *n* = 8) or supernatants of *P. faecium* DSM 32890 primed BMDMØ (MO-*P. faecium* *n* = 10) for 6 h. **h**, Cytokine levels in the ILC1 culture supernatant (*n* = 8). **i**, mRNA relative expression of indicated genes of intestinal ILC1 cultures challenged with *P. faecium* DSM 32890 for 6 h (*n* = 8). Values are presented as mean ± s.e.m. of *n* biological replicates shown as individual dots. Significant differences were assessed using unpaired Student’s *t*-test (two-sided), one-way ANOVA (**e**) or two-way ANOVA (**f**) followed by a post hoc Tukey’s test. *P < 0.05, **P < 0.01, *** *P* < 0.001 and ****P < 0.0001. Source data

We further investigated the nature of the interactions between *P. faecium*, macrophages and ILC1s in vitro (Fig. 6a). Flow cytometry analysis revealed that exposure of bone marrow-derived macrophages (BMDMØ) to *P. faecium* (1:10 cell/bacteria ratio) triggered a marked augmentation of M2 differentiation markers (Arg1, CD206 and CD163) (Fig. 6b) and an increased secretion of IL-10 and IL-1β (Fig. 6c), whereas interferon (IFN)γ, IL-22, IL-23, IL-12p70, granulocyte-macrophage colony-stimulating factor (GM-CSF) and IL-4 remained below the detection threshold. To identify the signalling pathway involved, we measured the changes in TLR gene expression. *P. faecium* upregulated *Tlr2* expression and decreased that of *Tlr4* and *Tlr5* (Fig. 6d). Then, we exposed HEK-Blue-hTLR2 cells to different *P. faecium* concentrations, confirming the interaction of the bacterium with TLR2 in a dose-response manner (Fig. 6e). Lastly, we demonstrated that the co-treatment of BMDMØ with *P. faecium* and an anti-TLR2 blunted the ability of *P. faecium* to increase M2 differentiation markers (Fig. 6f), showing causality.

Finally, we questioned whether the *P. faecium-*induced changes in ILC1s in obesity models were mediated by macrophages, by exposing intestinal ILC1 cultures to the supernatant of *P. faecium*-stimulated BMDMØ cultures. Results showed an increase in the gene expression of *Ifng, Tbx21, Il22* and *Il23r*, a decrease in *Rorgc* expression and no changes in *Tnfa* (Fig. 6g). We also detected markedly higher levels of IL-22 and a small decrease in IFNγ levels (*P* = 0.087) in ILC1 cultures exposed to supernatant of *P. faecium*-stimulated BMDMØ cultures (Fig. 6h). None of these effects were reproduced in ILC1 cultures directly exposed to *P. faecium* at a 1:10 cell/bacteria ratio (Fig. 6i). Altogether, these findings demonstrate the potential of *P. faecium* to polarize macrophages to the M2 phenotype through a TRL2-dependent mechanism, which in turn controls the inflammatory cascade seconded by ILC1s.

## Discussion

Here we demonstrate the anti-obesogenic properties of the strain *P. faecium* DSM 32890, an intestinal symbiont, and its mode of action on specific intestinal immune circuits. Our meta-analysis robustly supports the association of *P. faecium* with normal weight in a large multicohort study in humans^15,22^. We also show that *P. faecium* DSM 32890 suppresses HFHSD-induced body weight gain by 25% and adiposity by 35%, and improves glucose homeostasis independently of its viability in mice. These anti-obesogenic effects are comparable to those of semaglutide in mice, supporting the biological importance of our findings^25^. However, studies on females should be warranted to consider sex-associated effects since our findings are restricted to males. Here we also provided strong evidence supporting the idea that the metabolic benefits are primarily mediated by the ability of *P. faecium* to boost M2-macrophage polarization, which in turn blunts the HFHSD-driven expansion of pro-inflammatory ILC1s in the gut.

The gut immune system is constantly challenged by hypercaloric diets^26^. Chronic exposure to energy-dense foods triggers gut inflammation, which plays an important role in the lasting metabolic complications of obesity^27^. We show that an HFHSD stimulates the accumulation of pro-inflammatory induced IELs (TCRαβ^+^) and ILC1s, and increases the M1/M2 ratio in the small intestine, partly in line with other studies^28,29^. This represents an early pathogenic gut immune signature of obesity that, if sustained, becomes systemic and causes metabolic dysfunction^4^. While an obesogenic diet is the primary trigger, diet-induced microbiota alterations are also causally involved in the dysregulation of gut immunity and inflammation, as demonstrated by faecal transplant studies^9^. Notably, alterations in the gut immune system can be reversed, representing a potential therapeutic target for obesity^4,10^. The inhibition of intestinal pro-inflammatory macrophage (M1-type-like) infiltration in genetically engineered animals (Ccr2 knockout mice and tamoxifen-inducible models) or by the oral administration of anti-inflammatory drugs mitigates obesity-associated inflammation and metabolic dysregulation^8,10^. Comparable to these studies, we found that controlling the intestinal immune response to an energy-dense diet by *P. faecium* administration alleviated systemic HFHSD-triggered inflammation and prevented obesity development. Specifically*, P. faecium* prevented the increase in gut pro-inflammatory immune cells and dramatically reduced the plasma levels of inflammatory cytokines in the DIO model.

In WT HFHSD-fed mice, *P. faecium* stimulated an intestinal pro-tolerogenic and anti-inflammatory phenotype evidenced by increases in Tregs, natural (TCRγδ+) IELs and M2 macrophages, which are the potential underlying protective immune mediators. Our findings in *Rag1*^−/−^ mice revealed that adaptive immunity does not primarily mediate the beneficial effects of *P. faecium* on body weight gain and metabolic dysfunction. Instead changes in intestinal M2 macrophages and ILC1s seem to be the primary drivers. The role of macrophages in obesity has been studied in depth^4,5,8^, revealing the benefits of inducing M2 polarization to reverse obesity-associated inflammation^30^. Nevertheless, the evident ability of *P. faecium* to reduce the abundance of gut ILC1s by priming macrophages towards an M2 phenotype expands previous knowledge. ILCs are involved in tissue homeostasis, morphogenesis and regeneration^31^, but the role of gut-resident ILC1s in the regulation of metabolism and obesity is still largely unknown. The increased gut abundance of ILC1s (and T-bet^+^ ILC3 cells) was related to the reduction of IL-22 and the exacerbation of metabolic disease in a DIO murine model^12^. We recently reported that blocking the increase in intestinal ILC1s induced in mice by HFHSD prevents M1 macrophage skewing and reinforces the intestinal barrier, thus alleviating metabolic dysfunction^29^. Here we show that *P. faecium* reduces the abundance of intestinal ILC1s by priming macrophages towards an M2 phenotype, and thus also improves gut defence mechanisms (reflected by increased sIgA production and overexpression of AMPs and IL-22). Collectively, our results reveal a chain of immune events induced by *P. faecium* in obesity. The bacterium directly primes M2-macrophage polarization via TLR2 signalling. The involvement of this molecular pathway is supported by the effect of anti-TLR2 antibodies in blunting the *P. faecium* induction of M2 activation markers in in vitro macrophage cultures and the direct TLR2 activation in HEK-Blue cells. Other authors have also pointed to the role of TLR2 in mediating anti-inflammatory^32^ or anti-obesogenic^18^ effects. Here we have shown causal mechanistic links between the *P. faecium*-induced M2 increases via TLR2 and the reduction of the obesity-induced ILC1 increases, allowing resetting of the gut immune homeostasis in obesity. It is tempting to speculate that *P. faecium*-primed macrophages trigger the conversion of ILC1s into ILC3-like cells, which have high plasticity^33^. Supporting this, we observed that *P. faecium* reduced ILC1 abundance in vivo and increased the levels of IL-1β in BMDMØ cultures. IL-1β is a cytokine that, despite being well-known for its pro-inflammatory role, also participates in ILC1 to ILC3 differentiation^33^ and inflammation resolution^34^. In addition, the exposure to the supernatant of *P. faecium*-stimulated BMDMØ cultures promoted IL-22 production by intestinal ILC1s. This was not accompanied by an increase in *Rorgc* expression or a decrease in *Tbx21* expression in ILC1 cultures stimulated with conditioned medium, which are described to be characteristic of the switch between ILC1s and ILC3s^33,35^. Nonetheless, some authors have reported that during pulmonary infection of mice, natural killer cells (members of the ILC1 group) increase the production of IL-22 to promote host defence independently of RORγt levels^36,37^, suggesting that IL-22 production is not always tied to this transcription factor. Indeed, the mechanisms underlying the different transient states acquired by ILCs in response to environmental stimuli, such as bacteria, await clarification.

We show that *P. faecium*-induced M2 macrophages are critical in blocking the expansion of pro-inflammatory ILC1s, as GW2580 co-treatment not only inhibited the increase in M2 macrophages but also the reduction in ILC1s, ultimately leading to an important loss of the anti-obesogenic effects of *P. faecium* (cancelling 62% of body weight reduction and 60% of the improvements in glycaemic control). That these effects were not completely blocked by M2-macrophage inhibition probably indicates additional mechanisms that control the energy balance, which warrants further investigation. Co-administration of GW2580 also prevented the *P. faecium*-driven reductions in GIP in HFHSD-fed mice. GIP contributes to obesity development through anabolic effects, leading to fat accumulation and WAT inflammation characterized by elevations in pro-inflammatory chemokines and cytokines such as CCL2 and IL-6 (ref. ^38^). Obesity development can be attenuated by neutralizing antibodies to GIP or by GIP receptor antagonists^39,40^. In addition, GIP is known to impair the neuronal sensitivity of leptin, an important controller of energy homeostasis, under excessive caloric intake^41,42^. We also noted less WAT inflammation and fat expansion in animals treated with *P. faecium*, which was reverted in animals co-administered with GW2580. These findings suggest that M2-macrophage induction by *P. faecium* is crucial not only for the control of inflammation but also for adiposity. Specifically, mice co-treated with *P. faecium* and GW2580 maintained the HFHSD-driven overexpression of *Ccl2*, *Cd11c*, *Tnfa* and *Il6* in WAT. CCL2 mediates monocyte recruitment, M1 polarization and the upregulation of TNF-α and IL-6 in fat pads, contributing to the inflammatory milieu linked to obesity-associated comorbidities^43,44^. Also, conditional ablation of CD11c^+^ cells lowers the levels of inflammatory cytokines in WAT and rapidly improves glucose intolerance in DIO mice^45^. However, we cannot exclude the possibility that GW2580 has affected other tissues, accounting for some of the effects observed. In addition, absolute macrophage numbers and their polarization into a broader spectrum of activation states were not fully captured by the M1/M2 phenotype assessments. This might have limited understanding of the bacterial effects on total and other macrophage subpopulations.

We found that live *P. faecium*, but not the pasteurized bacterium, partly buffers the microbiota alterations caused by HFHSD. For example, live *P. faecium* administration enhanced the abundance of *A. muciniphila*, which theoretically could potentiate the metabolic benefits of live *P. faecium* in obesity^18,46^. *P. faecium*, which is a propionate and potential butyrate producer bacterium^47^, also favours the presence of butyrate and mucus producers (Ruminococcaceae*_*UBA1819 spp. and *Mucispirillum* spp.) in our study, which could play additional beneficial roles in strengthening the gut barrier^48^. Overall, our findings suggest that live *P. faecium* facilitates the restoration of a beneficial ecological niche under HFHSD feeding. Nonetheless, the inability of the pasteurized bacterium to modify the gut microbial structure indicates that this bacterium per se has immunoregulatory and anti-obesogenic properties. The preservation of the anti-obesogenic effects in pasteurized *P. faecium* combined with the in vitro findings suggests that a structural component, acting as a TLR2 ligand, is the primary driver of the direct macrophage’s modulation. Further research would be warranted to identify the specific bacterial motif responsible for the *P. faecium* effects.

Finally, our large multicohort study of stool metagenomes indicates that the species *P. faecium* is more prevalent in non-obese participants regardless of age and sex, which can be of potential value as an indicator of obesity. This result is in line with previous findings of small-scale studies^15,22^ and supports the role of *P. faecium* as a natural opposer to the insurgence of obesity. Furthermore, other species of the same genus show no association or opposite associations and no effects in vivo, supporting the specificity of the *P. faecium* link to normal weight in humans.

In conclusion, this study provides insights into the beneficial role of *P. faecium* in obesity and unravels the causal immune mechanism through which this bacterium may protect from obesity. *P. faecium* DSM 32890 resets the communication between macrophages and gut-resident ILCs, priming M2 polarization which buffers the hypercaloric diet-induced increase in ILC1s, ultimately reverting systemic inflammation and metabolic dysfunction. Overall, our results contribute to the identification of keystone bacteria of potential relevance to understanding the origin of obesity and the mechanisms through which they can intercept the pathogenic chain of events.

## Methods

### Meta-analysis of human metagenomes

To quantify differences in the probability of detecting *P. faecium* at a larger scale, we collected 7,569 human metagenomes from healthy adult individuals with metadata available in curatedMetagenomicData 3 (ref. ^23^). Samples were downloaded from NCBI and profiled taxonomically using MetaPhlAn 4 (Jun23 database)^49^. Using curated Metagenomic Data 3, public samples were annotated as adult healthy participants with reported BMI, age and sex. Using the scripts available at 10.5281/zenodo.15120683 (ref. ^50^), we built two datasets. The first comprised 4,050 individuals with a BMI < 25 (normal weight) and 2,532 individuals with BMI ≥ 25 (overweight). This dataset spanned 28 studies from 15 nationalities. The second totalled 3,652 individuals with BMI < 30 (non-obese) and 1,135 individuals with BMI ≥ 30 (obese) (8 nationalities and 15 studies). Studies with less than 15 individuals in the smallest group were excluded. We run a logistic regression model in each study in the two datasets assessing the relationship with the presence of *P. faecium* (defined as the non-zero abundance in MetaPhlAn 4), and we synthesized the resulting overall effects by random-effect meta-analysis with DerSimonian–Laird heterogeneity. In the first dataset, the individuals with BMI > 25 were considered the positive class and the negative coefficient is associated with lower BMI. In the second dataset, the positive class was constituted by the individuals with obesity. For the two datasets, we performed two additional analyses in each, by adjusting the logistic regression coefficients by either sex or age and performing an identical meta-analysis. In addition, we performed an identical meta-analysis of overweight versus normal-weight individuals on the species *P. succinatutens* and *P*. spp. ET69, which were the only *Phascolarctobacterium* species identified in the two datasets.

### Bacteria growth conditions

*P. faecium* DSM 32890 was isolated from the stool of a healthy volunteer^47^, and *P. succinatutens* DSM 22533 was obtained from the DSMZ collection. Bacteria were grown in modified PYG medium, replacing glucose with succinate (8 g l^−1^), and incubated at 37 °C under anaerobic conditions (80% N_2_, 10% CO_2_ and 10% H_2_) using a Bactron300-2 anaerobic chamber (Shel Lab). For animal experiments, the bacterial biomass was collected by centrifugation (10,000 × *g*, 10 min, 4 °C), washed twice with PBS (130 mM sodium chloride, 10 mM sodium phosphate, pH 7.4) supplemented with 0.05% l-cysteine (Sigma-Aldrich) and resuspended in PBS containing 0.05% l-cysteine and 10% glycerol. Bacterial stocks were stored at −80 °C until use. After freezing and thawing, the number of viable bacteria was calculated using BD Trucount tubes (Becton Dickinson) and propidium iodide staining (Sigma-Aldrich) in a BD LSRFortessa flow cytometer (Becton Dickinson). The absence of contamination and bacterium identity were routinely confirmed by Gram staining and 16S rRNA Sanger gene sequencing using the universal primers 27F (5′-AGAGTTTGATCCTGGCTCAG-3′) and 1401R (5′-CGGTGTGTACAAGACCC-3′). For pasteurization, *P. faecium* was heated at 70 °C for 30 min and then frozen, avoiding important temperature changes. To confirm the non-viability of pasteurized *P. faecium*, samples were plated into modified PYG agar plates and incubated for 72 h at 37 °C under anaerobic conditions.

### Animal experimental design

Seven-week-old C57BL/6J WT male mice were purchased from Charles River Laboratories. *Rag1*^−*/*−^ male mice with a C57BL/6J background were supplied by The Jackson Laboratory. Mice were randomly housed in groups of 4–5 animals per cage in a ventilated rack under controlled temperature (23 ± 2 °C) and relative humidity (40–50%), and with a 12-h light/dark cycle. Mice were acclimatized for 10 days and had ad libitum access to water and food. For experiments, mice were randomized on the basis of body weight to minimize baseline differences. Body weight evolution, and food and water intake were monitored twice weekly. In every experiment, animals received their daily bacterial doses at a consistent morning time. No adverse effects of treatment were observed.

#### Experiment 1

WT male mice (30) were divided into the following three experimental diet groups for 14 weeks (*n* = 10): (1) CD (D12450K, Ssniff; 10% of energy from fat and no sucrose), (2) HFHSD (D12451, Ssniff; 45% of energy from lard fat and 17% from sucrose) and (3) HFHSD plus an oral gavage of *P. faecium* (~2 × 10^9^ cells day^−1^) (HFHSD + *P. faecium*). The bacterium vehicle consisting of PBS supplemented with 0.05% l-cysteine and 10% glycerol was dosaged to control groups.

#### Experiment 2

WT male mice (15) were divided into three experimental groups: (1) HFHSD, (2) HFHSD + *P. faecium* and (3) HFHSD + *P. succinatutens*. Both bacteria were administered orally at an equal dose (~2 × 10^9^ cells day^−1^), while the control group received the corresponding vehicle during 8 weeks.

#### Experiment 3

Male *Rag1*^−/−^ mice (30) were divided into the following three experimental diet groups for 14 weeks: (1) CD, (2) HFHSD and (3) HFHSD + *P. faecium* (~2 × 10^9^ cells day^−1^). Control groups received the corresponding vehicle.

#### Experiment 4

Male WT mice (32) were divided into four experimental diet groups for 14 weeks: (1) CD, (2) HFHSD, (3) HFHSD + *P. faecium* and (4) HFHSD and a daily dose of pasteurized *P. faecium* (HFHSD + *P. faecium* pasteurized). Viable and pasteurized bacteria were administered orally (~2 × 10^9^ cells day^−1^). Control groups received the corresponding vehicle.

#### Experiment 5

Male WT mice (40) were administered (1) CD, (2) HFHSD, (3) HFHSD + *P. faecium* and (4) HFHSD + *P. faecium* + GW2580 (which inhibits colony stimulating factor 1 receptor phosphorylation) to prevent M2-macrophage polarization (HFHSD + *P. faecium* + GW2580). Bacteria were administered orally (~2 × 10^9^ cells day^−1^). For validation purposes, a fifth group was treated with HFHSD and the inhibitor (HFHSD + GW2580). The inhibitor was dissolved in PBS with 0.1% Tween 80 and 0.5% hydroxyl methyl cellulose, and was administered daily by oral gavage at 160 mg kg^−1^ day^−1^ (ref. ^51^), starting at week 4 after the introduction of the HFHSD until the end of the experiment. Control groups (1, 2) received the corresponding vehicle containing the inhibitor solvent and the bacterium vehicle. In addition, group (3) received a vehicle containing the inhibitor solvent, and group (4) received the bacterium vehicle.

At the end of the experiment, animals were fasted, anaesthetised with isoflurane and killed by cervical dislocation. The kill order was designed to minimize potential confounders, hence mice of the different experimental groups were interspersed. Blood was collected by cardiac puncture in EDTA-containing tubes. Plasma was immediately collected after centrifugation (1,000 × *g*, 5 min, 4 °C). Plasma, intestinal tissue, adipose tissue and caecal content were snap frozen in liquid nitrogen or kept on ice for flow cytometry analysis. All samples were stored at −80 °C until further use.

### Ethics

Animal procedures were evaluated and approved by the ethics committee of the University of Valencia (Animal Production Section, SCSIE, University of Valencia) and authorized by the competent authority (Generalitat Valenciana) which assigned the following approval IDs: 2017/VSC/PEA/00015, 2018/VSC/PEA/0171 and 2021/VSC/PEA/0177. The procedures conformed with EU directive 2010/63/UE and the Spanish RD53/2013 regulation regarding the protection of animals used for experimental and other scientific purposes.

### Oral glucose tolerance test

An oral glucose tolerance test was conducted 2–3 weeks before killing. Mice were fasted for 4 h before collecting blood from the saphenous vein at 0, 15, 30, 60 and 120 min in response to an oral glucose challenge (2 g kg^−1^ body weight). Glycaemia was determined using a Contour XT glucometer (Bayer).

### Isolation of immune cells and flow cytometry analysis

**Extended Data Fig. 4:**
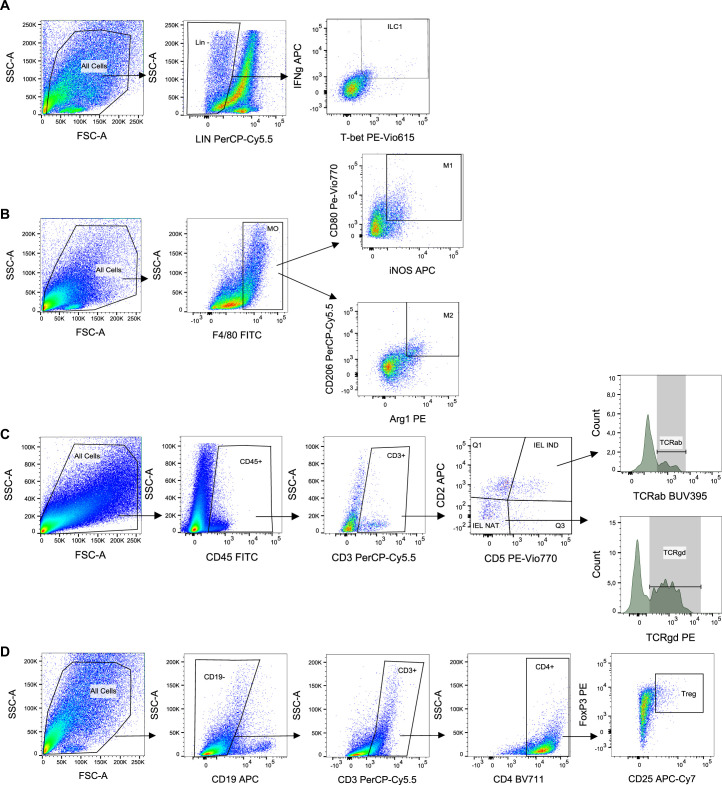
Gating strategy for flow cytometry analysis. After single-cell gating of different intestinal immune populations: (**a**) Innate lymphoid cells group 1 (ILC1) isolated from the small intestine epithelium were characterized as Lin-T-bet+ IFN-γ+ cells. Commercial BD PerCP-Cy™5.5 Mouse Lineage Antibody Cocktail was used for lineage containing: CD3ε, CD45R/B220, Ter119, Gr1,Ly6c, Cd11b. (**b**) Macrophages isolated from small intestine lamina propria were characterized as pro-inflammatory macrophages (M1): F4/80 + CD80+ iNOS+ cells and alternative activated macrophages (M2): F4/80 + CD206+ Arg1+ cells, (**c**) Intraepithelial lymphocytes (IELs) isolated from the small intestine epithelium were characterized as induced IELs: CD45 + CD3 + CD2 + CD5 + TCRαβ+ cells and natural IELs: CD45 + CD3 + CD2-CD5-TCRγδ+, (**d**) T regulatory cells (T reg) isolated from the small intestine lamina propria were characterized as: CD19-CD3 + CD4 + CD25+Foxp3+ cells.

Isolation and staining of intestinal immune cells was conducted as previously described^28,29^. Briefly, the small intestine was washed with ice-cold PBS, opened longitudinally and cut into small pieces. Intestinal tissue was incubated for 30 min at 37 °C in Hanks balanced salt solution (HBSS) (ThermoFisher) supplemented with 5 mM EDTA (Scharlab), 1 mM dithiothreitol (Sigma-Aldrich), 100 µg ml^−1^ streptomycin and 100 U ml^−1^ penicillin (P/S, Merck). Cells were passed through 100-µm nylon cell strainers (Biologix), washed in PBS supplemented with 5% fetal bovine serum (FBS, Mercodia) and centrifuged (300 × *g*, 5 min, 4 °C) to collect the intra-epithelial cell fraction. The remaining tissue in the cell strainer was incubated twice at 37 °C for 30 min in HBSS supplemented with 0.5 mg ml^−1^ collagenase D (Roche Diagnostics ), 3 mg ml^−1^ dispase II (Sigma-Aldrich), 50 U ml^−1^ DNase I (Roche Diagnostics) and P/S. Cells from the lamina propria were collected by centrifugation after filtration of the supernatant with 70-µm nylon cell strainers (Biologix) and washing in PBS supplemented with 5% FBS. We characterized the following three immune cell subsets. In the intestinal epithelium: ILC1s: Lin^−^T-bet^+^ IFN-γ^+^; induced IELs: CD45^+^CD3^+^CD2^+^CD5^+^ TCRαβ^+^; and natural IELs: CD45^+^CD3^+^CD2^−^CD5^−^ TCRγδ^+^. In the lamina propria, we analysed pro-inflammatory macrophages (M1): F4/80^+^CD80^+^ iNOS^+^; alternative activated macrophages (M2): F4/80^+^CD206^+^ Arg1^+^; and regulatory T (Treg) cells: CD19^−^CD3^+^CD4^+^CD25^+^ Foxp3^+^. For lineage analysis, we used the commercial BD PerCP-Cy5.5 Mouse Lineage Antibody Cocktail. All the antibodies used for flow cytometry analyses and the gating strategy followed are detailed in Supplementary Table 4 and Extended Data Fig. 4. In all cases, for the detection of intracellular markers, cells were first permeabilized and fixed (fixation/permeabilization solution kit, BD Bioscience). Data were acquired with a BD LSRFortessa flow cytometer operated with FACS Diva software v.7.0 (BD Biosciences) and analysed using FCS express v.5 flow cytometry software or FACS Diva software v.7.0.

#### GM-CSF bone marrow-derived cell generation and stimulation

**Extended Data Fig. 5:**
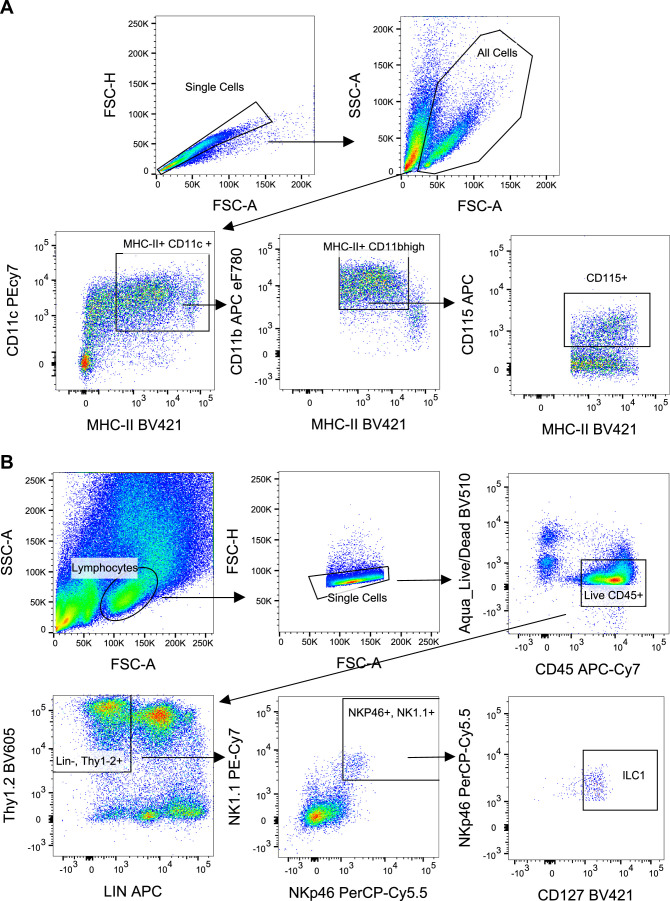
Gating strategy for flow cytometry analysis and cell sorting. (**a**) Bone marrow-derived macrophages (BMDMØ) were characterized as CD11c+CD11b^high^MHC-II + CD115+ cells and polarization to M2 was determined by the expression of Arg-1, CD206 and/or CD163. (**b**) Sorted Innate lymphoid cells group 1 (ILC1) isolated from the small intestine were defined as CD45.2+Lin-CD127 + CD90.2 + NK1.1 + NKp46+ cells. The lineage cocktail included: CD3ε, CD8a, CD19, Ter119, Cd11c, TCRb, TCRgd, Gr1, Cd11b.

Isolation and differentiation of murine bone marrow-derived cells were performed on the basis of a published protocol^52^ with slight modifications. Briefly, bone marrow cells were extracted from the femur and tibia of 4–7-week-old C57BL/6 mice by flushing with RPMI-1640 medium. Cells were filtered using a 70-μm strainer (Biologix) and collected (300 × *g*, 5 min, 4 °C). Cells were first cultured on Petri dishes in RPMI medium containing 10% FBS, 1% P/S and 20 ng ml^−1^ GM-CSF (PeproTech) under microaerobic conditions (5% CO_2_) at 37 °C. On day 3, the medium was refreshed, and on day 6 the medium was removed and the BMDMØ were detached using Accutase (Sigma-Aldrich). Cells were pelleted and resuspended in RPMI medium with 10% FBS and plated in 6-well plates (1 × 10^6^ cells per well). The next day, BMDMØ were exposed for 6 h to *P. faecium* at a ratio of 1:10 cell/bacteria, to anti-TLR2 (clone C9A12; MABG-MTLR2-2, InvivoGen) at 1 μg ml^−1^ or both. Anti-TLR2 was pre-incubated for 1 h with the cells in advance of additional stimuli following manufacturer specifications. The conditioned medium was collected for cytokine quantification and ILC1 stimulation, while 0.1 ml of the supernatant was inoculated in modified PYG agar to confirm the viability of the bacteria by plate counting. BMDMØ cells were used either for flow cytometry or RNA extraction. Challenged BMDMØ cells were incubated with anti-mouse FcgRIII/II CD16/CD32 before staining. Macrophages were characterized as CD11c^+^CD11b^high^MHC-II^+^CD115^+^ cells, and polarization to M2 was determined by the expression of Arg1, CD206 and/or CD163, as described elsewhere^52^. The antibodies used and the gating strategy followed are detailed in Supplementary Table 4 and Extended Data Fig. 5a. The cells used for RNA extraction were homogenized and stored at −80 °C in a lysis buffer (Qiagen) until used. Two independent experiments were performed with at least 6 biological replicates per condition.

#### Culture and stimulation of human HEK293-Blue hTLR2 cells

HEK-Blue hTLR2 cell lines (HEK293 cells; hkb-htlr2, Invivogen) were used for TLR2 stimulation analysis. Cells were grown and subcultured following the manufacturer’s procedure in Dulbecco’s modified Eagle medium (DMEM) supplemented with 4.5 g l^−1^ d-glucose, 50 U ml^−1^ penicillin, 50 μg ml^−1^ streptomycin, 100 μg ml^−1^ normocin, 2 mM l-glutamine, 10% (v/v) heat-inactivated FBS and HEKs-Blue selection antibiotics. The immune response experiment was carried out by seeding HEK-Blue cells in flat-bottom 96-well plates with HEK-Blue detection media and stimulating them by the addition of 20 μl *P. faecium* at decreasing concentrations from 10^8^ to 10^5^ bacterial cells per ml. The 96-well plates were incubated for 16 h at 37 °C in a 5% CO_2_ incubator. TLR2 ligand FSL-1 at 1 ng ml^−1^ was used as positive control, whereas maintenance medium (DMEM) without any selective antibiotics was used as negative control. Secreted alkaline phosphatase was detected by measuring the optical density at 655 nm. Three independent experiments were performed with 6 technical replicates per condition.

#### ILC1 isolation, purification and co-culture

ILC1s were isolated from the lamina propria of the small intestine of 9–12-week-old C57BL/6 mice and purified by fluorescence-activated cell sorting on the Aria Cell sorter (Becton Dickinson Biosciences) as described elsewhere^53^. Isolated cells were incubated with anti-mouse FcgRIII/II CD16/CD32 before staining, and the ILC1 population was gated for live cells defined as: CD45.2^+^Lin^−^CD127^+^CD90.2^+^NK1.1^+^NKp46^+^. The lineage cocktail included CD3ε, CD8a, CD19, Ter119, Cd11c, TCRb, TCRgd, Gr1 and Cd11b^53,54^. Antibody details and the gating strategy followed can be found in Supplementary Table 4 and Extended Data Fig. 5b. After sorting, cells were plated in 96-well plates (2.5 × 10^3^ cells per well) and incubated at 5% CO_2_ and 37 °C with murine IL-2, IL-7 and IL-12 (all at a final concentration of 10 ng ml^−1^) in complete RPMI medium containing RPMI-1640, 10% FBS, 0.1% 2-mercaptoethanol (50 mM), 2 mM l-glutamine, 100 U penicillin, 0.1 mg ml^−1^ streptomycin, 2.5 mM HEPES, 1 mM sodium pyruvate and 1 mM MEM non-essential amino acids. On the next day, ILC1s were exposed to *P. faecium* in a cell/bacteria ratio of 1:10 or to *P. faecium*-stimulated BMDMØ conditioned medium, for 6 h. The conditioned medium was used for cytokine quantification and cells were homogenized in a lysis buffer (Macherey-Nagel) for RNA extraction. Two independent experiments were performed with 4–5 biological replicates per condition.

### Biochemical and immune parameters

Fasting glycaemia was determined in blood plasma using the Contour XT glucometer before killing the animal. Plasma triglycerides and cholesterol were quantified with the triglyceride colorimetric assay kit (Elabscience) and the cholesterol liquid kit (Química Analítica Aplicada). GIP, insulin and leptin were quantified using the Luminex Mouse Metabolic Hormone Expanded kit (Merck). Insulin resistance was calculated using the homeostatic model assessment for insulin resistance (HOMA-IR) index as fasting plasma insulin (mU l^−1^) × fasting plasma glucose (mmol l^−1^)/22.5. Plasma levels of IFN-γ, IL-6, IL-1α, IL-1β, TNFα, IL-23, IL-27 and IL-10 were determined using the LEGENDplex mouse inflammation panel (Biolegend), and circulating levels of LBP were determined with the corresponding enzyme-linked immunosorbent assay kit (Cloud-clone). IL-1β, IL-10, IFNγ, IL-22, IL-23, IL-12p70, GM-CSF and IL-4 were quantified in BMDMØ supernatants. IFNγ and IL-22 levels were measured in the supernatants of the ILC1 cultures. In both cases we used a Milliplex mouse Th17 magnetic bead panel (Merck). Before measuring sIgA, the caecal content was disaggregated in PBS (100 mg ml^−1^), homogenized using Lysing Matrix D tubes (MP Biomedicals) and centrifuged (1,000 × *g*, 5 min, 4 °C). The supernatant was diluted (1:1,000) with assay buffer and sIgA was quantified using a commercial ELISA kit (Invitrogen).

### Gene expression

RNA isolation from the small intestine was performed using the TRIsure reagent (Bioline), as described elsewhere^14^. RNA concentration was determined using the Nanodrop 2000c spectrophotometer (ThermoFisher). For each reaction, 1–2 μg of total RNA was reverse transcribed to cDNA using the High-Capacity cDNA Reverse Transcription kit (Applied Biosystems). RT–qPCR was performed on the LightCycler 480 Instrument (Roche). The reaction consisted of LightCycler 480 SYBR Green I Master Mix (Roche) and 300 nM of gene-specific primer pairs. For cultured BMDMØ and ILC1s, RNA was extracted using the RNeasy Plus mini kit (Qiagen) or the NucleoSpin RNA XS (Macherey-Nagel), respectively. RNA was retrotranscribed using a High-Capacity RNA-to-cDNA kit (Applied Biosystems), followed by a pre-amplification PCR using *Taq*Man PreAmp Master Mix (Applied Biosystems). RT–qPCR was performed with *Taq*Man Gene Expression Master Mix using a QuantStudio 5 Real-Time PCR system (Applied Biosystems). Primers and probe sequences can be found in Supplementary Table 5. In all cases, samples were run in duplicate and the data were analysed using the comparative $2^{-\Delta\Delta C_{\rm{T}}}$ method. Target genes were normalized with *Rpl19, Gapdh* or *Hprt* housekeeping genes and reported as mRNA fold change compared to the CD group.

### Gut microbiota analysis

DNA from the caecal content of mice was extracted using the QIAmp Fast DNA Stool mini kit (Qiagen). Library preparation was performed using the Nextera XT v.2 Index (Illumina) targeting the V3–V4 region of the 16S rRNA gene and sequenced on an Illumina MiSeq platform (2 × 300 bp paired-end reads). Quantification of ASVs was performed with the DADA2 v.1.24 R package^55^. Raw reads were truncated after 250 bp for forward and reverse reads, and 30 nucleotides were additionally removed at the start of both paired-end reads. Reads were filtered for quality assurance; clean pairs of reads were merged into contig sequences and chimaeric sequences were discarded. Taxonomy was assigned by checking sequences against the SILVA v.138 database^56^. Taxa with a prevalence below 5% were removed from subsequent analyses. Data were rarefied according to the minimum number of lectures in the matrix 33,935. Alpha diversity was calculated by estimating the observed ASVs, Shannon index and Inverse Simpson index using the Phyloseq v.1.40 R package^57^. Non-parametric methods such as Kruskal–Wallis and post hoc Wilcoxon rank-sum tests were applied for statistical analysis of alpha diversity. Evaluation of the community structure across groups was performed through principal coordinate analysis (phyloseq::ordinatefunction and ‘weighted UniFrac’ distance). The analysis of the differential abundance of microbial taxa was performed using the DESeq2 v.1.36 R package^58^. The DESeq2-implemented function normalized our data and performed hypothesis testing using the Wald test. The resulting *P* values were corrected using the Benjamini–Hochberg false discovery rate (FDR) procedure.

### Statistical analysis

Statistical methods were not used to predetermine sample sizes, but our sample sizes are similar to those reported in previous publications^28,29^. Grubbs’ test was used for outlier detection, and the Shapiro–Wilk test was employed to assess data normality. Differences were determined using *t*-test or by one- or two-way analysis of variance (ANOVA) (as suitable), followed by Tukey’s post hoc multiple comparisons test when normally distributed. Kruskal–Wallis test followed by Dunn’s multiple comparisons test was used to analyse non-normally distributed data. The results are shown as mean ± s.e.m. and *n* represents the number of biological replicates shown as individual dots. Differences at *P* < 0.05 were considered significant and are represented using asterisks (*) unless otherwise stated. Data analysis was not blinded.

### Reporting summary

Further information on research design is available in the Nature Portfolio Reporting Summary linked to this article.

## Supplementary information

Supplementary Information Supplementary Tables 1–5. Due to Table 3 extension, an additional Supplementary Table 3 (Excel) has been uploaded with this data table.

Reporting Summary

Supplementary Table 3 Data for Supplementary Table 3.

## Source data

Source Data Fig. 2 Statistical source data.

Source Data Fig. 3 Statistical source data.

Source Data Fig. 4 Statistical source data.

Source Data Fig. 5 Statistical source data.

Source Data Fig. 6 Statistical source data.

Source Data Extended Data Fig. 2 Statistical source data.

Source Data Extended Data Fig. 3 Statistical source data.

## Data Availability

Data supporting the findings of this study are found in the source data files. The sequencing data corresponding to the murine microbiota analysed in this study have been deposited in the European Nucleotide Archive (ENA) at EMBL-EBI under accession number PRJEB59864 (https://www.ebi.ac.uk/ena/browser/view/PRJEB59864). For human studies, we used human metagenomes from healthy adult individuals with metadata available at https://waldronlab.github.io/curatedMetagenomicData/. Source data are provided with this paper.

## Code availability

The script data used to create the two human databases are available on GitHub at https://github.com/waldronlab/curatedMetagenomicDataAnalyses/blob/main/python_tools/meta_analysis_data.py (ref. ^50^).

The script data used for the meta-analyses in the human studies are available on GitHub at https://github.com/SegataLab/inverse_var_weight/blob/main/meta_analyses.py (ref. ^50^).

The script data corresponding to the murine microbiota analysed in this study are available on GitHub at https://github.com/INNOBIOME/Macrophages_Pfaecium_MicrobiotaAnalysis (ref. ^59^).

## References

[1] World Health Organization. Obesity and overweight. *Fact sheets* https://www.who.int/news-room/fact-sheets/detail/obesity-and-overweight (accessed 24 January 2023).

[2] Zhou, H., Urso, C. & Jadeja, V. Saturated fatty acids in obesity-associated inflammation. *J. Inflamm. Res.* **13**, 1–14 (2020). 32021375 10.2147/JIR.S229691 PMC6954080

[3] Amabebe, E. et al. Microbial dysbiosis-induced obesity: role of gut microbiota in homoeostasis of energy metabolism. *Br. J. Nutr.* **123**, 1127–1137 (2020). 32008579 10.1017/S0007114520000380

[4] Khan, S. et al. Emerging concepts in intestinal immune control of obesity-related metabolic disease. *Nat. Commun.* **12**, 2598 (2021). 33972511 10.1038/s41467-021-22727-7 PMC8110751

[5] Rohm, T. V. et al. Obesity in humans is characterized by gut inflammation as shown by pro-inflammatory intestinal macrophage accumulation. *Front. Immunol.* **12**, 668654 (2021). 34054838 10.3389/fimmu.2021.668654 PMC8158297

[6] Yudanin, N. A. et al. Spatial and temporal mapping of human innate lymphoid cells reveals elements of tissue specificity. *Immunity* **50**, 505–519.e4 (2019). 30770247 10.1016/j.immuni.2019.01.012 PMC6594374

[7] Monteiro-Sepulveda, M. et al. Jejunal T cell inflammation in human obesity correlates with decreased enterocyte insulin signaling. *Cell Metab.* **22**, 113–124 (2015). 26094890 10.1016/j.cmet.2015.05.020

[8] Kawano, Y. et al. Colonic pro-inflammatory macrophages cause insulin resistance in an intestinal Ccl2/Ccr2-dependent manner. *Cell Metab.* **24**, 295–310 (2016). 27508875 10.1016/j.cmet.2016.07.009

[9] Garidou, L. et al. The gut microbiota regulates intestinal CD4 T cells expressing RORγt and controls metabolic disease. *Cell Metab.* **22**, 100–112 (2015). 26154056 10.1016/j.cmet.2015.06.001

[10] Luck, H. et al. Regulation of obesity-related insulin resistance with gut anti-inflammatory agents. *Cell Metab.* **21**, 527–542 (2015). 25863246 10.1016/j.cmet.2015.03.001

[11] Tilg, H. et al. The intestinal microbiota fuelling metabolic inflammation. *Nat. Rev. Immunol.* **20**, 40–54 (2020). 31388093 10.1038/s41577-019-0198-4

[12] Okamura, T. et al. Trans fatty acid intake induces intestinal inflammation and impaired glucose tolerance. *Front. Immunol.* **12**, 669672 (2021). 33995404 10.3389/fimmu.2021.669672 PMC8117213

[13] Zou, J. et al. Fiber-mediated nourishment of gut microbiota protects against diet-induced obesity by restoring IL-22-mediated colonic health. *Cell Host Microbe* **23**, 41–53.e4 (2018). 29276170 10.1016/j.chom.2017.11.003 PMC6005180

[14] Liébana-García, R. et al. The allium derivate propyl propane thiosulfinate exerts anti-obesogenic effects in a murine model of diet-induced obesity. *Nutrients* **14**, 440 (2022). 35276798 10.3390/nu14030440 PMC8839906

[15] Rampelli, S. et al. Pre-obese children’s dysbiotic gut microbiome and unhealthy diets may predict the development of obesity. *Commun. Biol.* **1**, 222 (2018). 30534614 10.1038/s42003-018-0221-5 PMC6286349

[16] Muscogiuri, G. et al. Gut microbiota: a new path to treat obesity. *Int. J. Obes. Supp.* **9**, 10–19 (2019). 10.1038/s41367-019-0011-7 PMC6683132 31391921

[17] Romaní-Pérez, M. et al. *Holdemanella biformis* improves glucose tolerance and regulates GLP-1 signaling in obese mice. *FASEB J.* **35**, e21734 (2021). 34143451 10.1096/fj.202100126R

[18] Plovier, H. et al. A purified membrane protein from *Akkermansia muciniphila* or the pasteurized bacterium improves metabolism in obese and diabetic mice. *Nat. Med.* **23**, 107–113 (2017). 27892954 10.1038/nm.4236

[19] Yoon, H. S. et al. *Akkermansia muciniphila* secretes a glucagon-like peptide-1-inducing protein that improves glucose homeostasis and ameliorates metabolic disease in mice. *Nat. Microbiol.* **6**, 563–573 (2021). 33820962 10.1038/s41564-021-00880-5

[20] Wu, F. et al. *Phascolarctobacterium faecium* abundant colonization in human gastrointestinal tract. *Exp. Ther. Med.* **14**, 3122–3126 (2017). 28912861 10.3892/etm.2017.4878 PMC5585883

[21] Naderpoor, N. et al. Faecal microbiota are related to insulin sensitivity and secretion in overweight or obese adults. *J. Clin. Med.* **8**, 452 (2019). 30987356 10.3390/jcm8040452 PMC6518043

[22] Muñiz Pedrogo, D. A. et al. Gut microbial carbohydrate metabolism hinders weight loss in overweight adults undergoing lifestyle intervention with a volumetric diet. *Mayo Clin. Proc.* **93**, 1104–1110 (2018). 30077203 10.1016/j.mayocp.2018.02.019 PMC6107068

[23] Pasolli, E. et al. Accessible, curated metagenomic data through ExperimentHub. *Nat. Methods* **14**, 1023–1024 (2017). 29088129 10.1038/nmeth.4468 PMC5862039

[24] Klinkert, K. et al. Selective M2 macrophage depletion leads to prolonged inflammation in surgical wounds. *Eur. Surg. Res.* **58**, 109–120 (2017). 28056458 10.1159/000451078

[25] Gabery, S. et al. Semaglutide lowers body weight in rodents via distributed neural pathways. *JCI Insight* **5**, e133429 (2020). 32213703 10.1172/jci.insight.133429 PMC7213778

[26] Liébana-García, R. et al. The gut microbiota as a versatile immunomodulator in obesity and associated metabolic disorders. *Best Pract. Res. Clin. Endocrinol. Metab.* **35**, 101542 (2021). 33980476 10.1016/j.beem.2021.101542

[27] Rohm, T. V. et al. Inflammation in obesity, diabetes, and related disorders. *Immunity* **55**, 31–55 (2022). 35021057 10.1016/j.immuni.2021.12.013 PMC8773457

[28] López-Almela, I. et al. *Bacteroides uniformis* combined with fiber amplifies metabolic and immune benefits in obese mice. *Gut Microbes* **13**, 1–20 (2021). 33499721 10.1080/19490976.2020.1865706 PMC8018257

[29] Liébana-García, R. et al. Intestinal group 1 innate lymphoid cells drive macrophage-induced inflammation and endocrine defects in obesity and promote insulinemia. *Gut Microbes* **15**, 2181928 (2023). 36823075 10.1080/19490976.2023.2181928 PMC9980552

[30] Ying, W. et al. MiR-690, an exosomal-derived miRNA from M2-polarized macrophages, improves insulin sensitivity in obese mice. *Cell Metab.* **33**, 781–790.e5 (2021). 33450179 10.1016/j.cmet.2020.12.019 PMC8035248

[31] Vivier, E. et al. Innate lymphoid cells: 10 years on. *Cell* **174**, 1054–1066 (2018). 30142344 10.1016/j.cell.2018.07.017

[32] Ren, C. et al. Identification of TLR2/TLR6 signalling lactic acid bacteria for supporting immune regulation. *Sci. Rep.* **6**, 34561 (2016). 27708357 10.1038/srep34561 PMC5052581

[33] Bernink, J. H. et al. Interleukin-12 and −23 control plasticity of CD127+ Group 1 and Group 3 innate lymphoid cells in the intestinal lamina propria. *Immunity* **43**, 146–160 (2015). 26187413 10.1016/j.immuni.2015.06.019

[34] Giesbrecht, K. et al. IL-1β as mediator of resolution that reprograms human peripheral monocytes toward a suppressive phenotype. *Front. Immunol.* **8**, 899 (2017). 28824627 10.3389/fimmu.2017.00899 PMC5540955

[35] Bal, S. M., Golebski, K. & Spits, H. Plasticity of innate lymphoid cell subsets. *Nat. Rev. Immunol.* **20**, 552–565 (2020). 32107466 10.1038/s41577-020-0282-9

[36] Xu, X. et al. Conventional NK cells can produce IL-22 and promote host defense in *Klebsiella pneumoniae* pneumonia. *J. Immunol.* **192**, 1778–1786 (2014). 24442439 10.4049/jimmunol.1300039 PMC3995347

[37] Kumar, P. et al. IL-22 from conventional NK cells is epithelial regenerative and inflammation protective during influenza infection. *Mucosal Immunol.* **6**, 69–82 (2013). 22739232 10.1038/mi.2012.49 PMC3835350

[38] Gögebakan, Ö. et al. GIP increases adipose tissue expression and blood levels of MCP-1 in humans and links high energy diets to inflammation: a randomised trial. *Diabetologia* **58**, 1759–1768 (2015). 25994074 10.1007/s00125-015-3618-4

[39] Boylan, M. O. et al. Gastric inhibitory polypeptide immunoneutralization attenuates development of obesity in mice. *Am. J. Physiol. Endocrinol. Metab.* **309**, E1008–E1018 (2015). 26487006 10.1152/ajpendo.00345.2015

[40] Killion, E. A. et al. Anti-obesity effects of GIPR antagonists alone and in combination with GLP-1R agonists in preclinical models. *Sci. Transl. Med.* **10**, eaat3392 (2018). 30567927 10.1126/scitranslmed.aat3392

[41] Kaneko, K. et al. Gut-derived GIP activates central Rap1 to impair neural leptin sensitivity during overnutrition. *J. Clin. Invest.* **129**, 3786–3791 (2019). 31403469 10.1172/JCI126107 PMC6715359

[42] Obradovic, M. et al. Leptin and obesity: role and clinical implication. *Front. Endocrinol.* **12**, 585887 (2021). 10.3389/fendo.2021.585887 PMC8167040 34084149

[43] Gschwandtner, M., Derler, R. & Midwood, K. S. More than just attractive: how CCL2 influences myeloid cell behavior beyond chemotaxis. *Front. Immunol.* **10**, 2759 (2019). 31921102 10.3389/fimmu.2019.02759 PMC6923224

[44] Kusminski, C. M., Bickel, P. E. & Scherer, P. E. Targeting adipose tissue in the treatment of obesity-associated diabetes. *Nat. Rev. Drug Discov.* **15**, 639–660 (2016). 27256476 10.1038/nrd.2016.75

[45] Patsouris, D. et al. Ablation of CD11c-positive cells normalizes insulin sensitivity in obese insulin resistant animals. *Cell Metab.* **8**, 301–309 (2008). 18840360 10.1016/j.cmet.2008.08.015 PMC2630775

[46] Depommier, C. et al. Supplementation with *Akkermansia muciniphila* in overweight and obese human volunteers: a proof-of-concept exploratory study. *Nat. Med.* **25**, 1096–1103 (2019). 31263284 10.1038/s41591-019-0495-2 PMC6699990

[47] Rachek, S. et al. Complete genome sequence of *Phascolarctobacterium faecium* G 104, isolated from the stools of a healthy lean donor. *Microbiol. Resour. Announc.* **10**, e01054-20 (2021). 33509983 10.1128/MRA.01054-20 PMC7844068

[48] Chen, J. & Vitetta, L. The role of butyrate in attenuating pathobiont-induced hyperinflammation. *Immune Netw.* **20**, e15 (2020). 32395367 10.4110/in.2020.20.e15 PMC7192831

[49] Blanco-Míguez, A. et al. Extending and improving metagenomic taxonomic profiling with uncharacterized species using MetaPhlAn 4. *Nat. Biotechnol.* **41**, 1633–1644 (2023). 36823356 10.1038/s41587-023-01688-w PMC10635831

[50] Manghi, P. SegataLab/inverse_var_weight: v1.0.0 (v1.0.0). *Zenodo*10.5281/zenodo.15120683 (2025).

[51] Conway, J. G. et al. Inhibition of colony-stimulating-factor-1 signaling in vivo with the orally bioavailable cFMS kinase inhibitor GW2580. *Proc. Natl Acad. Sci. USA* **102**, 16078–16083 (2005). 16249345 10.1073/pnas.0502000102 PMC1276040

[52] Hickey, A. et al. *Bifidobacterium breve* exopolysaccharide blocks dendritic cell maturation and activation of CD4+ T cells. *Front. Microbiol.* **12**, 653587 (2021). 34220742 10.3389/fmicb.2021.653587 PMC8242212

[53] Godinho-Silva, C. et al. Light-entrained and brain-tuned circadian circuits regulate ILC3s and gut homeostasis. *Nature* **574**, 254–258 (2019). 31534216 10.1038/s41586-019-1579-3 PMC6788927

[54] Xu, W. et al. NFIL3 orchestrates the emergence of common helper innate lymphoid cell precursors. *Cell Rep.* **10**, 2043–2054 (2015). 25801035 10.1016/j.celrep.2015.02.057

[55] Callahan, B. J. et al. DADA2: high-resolution sample inference from Illumina amplicon data. *Nat. Methods* **13**, 581–583 (2016). 27214047 10.1038/nmeth.3869 PMC4927377

[56] Quast, C. et al. The SILVA ribosomal RNA gene database project: improved data processing and web-based tools. *Nucleic Acids Res.* **41**, D590–D596 (2013). 23193283 10.1093/nar/gks1219 PMC3531112

[57] McMurdie, P. J. & Holmes, S. phyloseq: an R package for reproducible interactive analysis and graphics of microbiome census data. *PLoS ONE* **8**, e61217 (2013). 23630581 10.1371/journal.pone.0061217 PMC3632530

[58] Love, M. I., Huber, W. & Anders, S. Moderated estimation of fold change and dispersion for RNA-seq data with DESeq2. *Genome Biol.* **15**, 550 (2014). 25516281 10.1186/s13059-014-0550-8 PMC4302049

[59] Rubio, T. & Sanz Lab. INNOBIOME/Macrophages_Pfaecium_MicrobiotaAnalysis. *Github* https://github.com/INNOBIOME/Macrophages_Pfaecium_MicrobiotaAnalysis (2025).

