# Supplementary material for: Gut commensal Phascolarctobacterium faecium retunes innate immunity to mitigate obesity and metabolic disease in mice
Source: Nat Microbiol. 2025 May 6;10(6):1310–22. doi: 10.1038/s41564-025-01989-7 (PMC12137122; doi:10.1038/s41564-025-01989-7)
Supplement: Supplementary file 1 — Supplementary Tables 1–5. Due to Table 3 extension, an additional Supplementary Table 3 (Excel) has been uploaded with this data table. [file 41564_2025_1989_MOESM1_ESM.pdf]

nature microbiology

Supplementary information

https://doi.org/10.1038/s41564-025-01989-7

Gut commensal *Phascolarctobacterium faecium* retunes innate immunity to mitigate obesity and metabolic disease in mice

In the format provided by the authors and unedited

Supplementary Table 1. Meta-analysis of association of *P. faecium* in a large-scale integrative cohort of human gut microbiomes from normal weight and overweight individuals.

	Effect	se	p	n over	n norm	pos over	pos norm
AsnicarF_2021_USA	-0.329	0.412	0.425	43	54	18	27
AsnicarF_2021_GBR	-0.228	0.131	0.081	388	612	162	290
CosteaPI_2017_KAZ	-0.412	0.479	0.389	51	33	14	12
FengQ_2015_AUT	-0.482	0.663	0.467	41	20	7	5
HMP_2012_USA	-0.981	0.438	0.025	35	60	15	40
JieZ_2017_CHN	-0.438	0.452	0.333	34	122	25	99
KarlssonFH_2013_SWE	-0.203	0.631	0.748	19	24	7	10
LeChatelierE_2013_DNK	-0.028	0.417	0.947	106	57	20	11
LifeLinesDeep_2016_NLD	-0.027	0.148	0.855	415	720	92	163
MetaCardis_2020_a_FRA	-0.547	0.316	0.083	83	93	26	41
MetaCardis_2020_a_DEU	-0.544	0.281	0.052	180	93	42	32
NagySzakalD_2017_USA	-0.094	0.963	0.923	19	31	17	28
NielsenHB_2014_DNK	-0.237	0.403	0.556	111	66	18	13
NielsenHB_2014_ESP	0.430	0.593	0.468	19	40	7	11
QinJ_2012_CHN	0.857	0.643	0.182	34	140	31	114
RubelMA_2020_CMR	-0.663	0.822	0.420	24	60	2	9
SchirmerM_2016_NLD	-0.836	0.453	0.065	44	412	6	110
XieH_2016_GBR	0.070	0.311	0.823	71	106	30	43
VogtmannE_2016_USA	0.662	0.607	0.275	17	35	8	11
WirbelJ_2018_DEU	-1.172	0.634	0.064	21	44	4	19
XuQ_2021_CHN	0.201	0.433	0.643	46	171	38	136
YachidaS_2019_JPN	-0.017	0.397	0.966	29	221	13	100
ZeeviD_2015_ISR	-0.011	0.142	0.936	385	464	145	176
ZellerG_2014_FRA	-0.249	0.668	0.709	18	41	4	11
ZhangX_2015_CHN	-0.394	0.536	0.462	24	69	17	54
iMSMS_2022_USA	-0.218	0.247	0.379	149	128	54	53
iMSMS_2022_ARG	0.190	0.400	0.635	60	69	17	17
iMSMS_2022_GBR	-0.430	0.457	0.347	66	65	10	14
Summary	-0.173	0.059	0.004	2532	4050	849	1649

Supplementary Table 2. Meta-analysis of association of *P. faecium* in a large-scale integrative cohort of human gut microbiomes from non-obese and obese individuals.

	**Effect**	**se**	**p**	**n over**	**n norm**	**pos over**	**pos norm**
AsnicarF_2021_USA	-0.437	0.562	0.437	16	81	6	39
AsnicarF_2021_GBR	-0.102	0.182	0.576	144	856	62	390
CosteaPI_2017_KAZ	-0.324	0.548	0.555	23	61	6	20
FengQ_2015_AUT	-0.555	0.730	0.447	21	40	3	9
HansenLBS_2018_DNK	-0.887	0.611	0.146	20	38	5	17
LeChatelierE_2013_DNK	0.020	0.404	0.960	94	69	18	13
LifeLinesDeep_2016_NLD	0.160	0.212	0.451	136	999	34	221
MetaCardis_2020_a_FRA	-0.630	0.321	0.049	77	99	23	44
MetaCardis_2020_a_DEU	-0.599	0.280	0.032	179	94	41	33
NielsenHB_2014_DNK	-0.211	0.396	0.594	99	78	16	15
XieH_2016_GBR	0.194	0.398	0.626	31	146	14	59
ZeeviD_2015_ISR	-0.289	0.176	0.101	184	665	60	261
iMSMS_2022_USA	-0.327	0.301	0.277	64	213	21	86
iMSMS_2022_ARG	0.134	0.531	0.801	21	108	6	28
iMSMS_2022_GBR	-0.258	0.598	0.666	26	105	4	20
Summary	-0.209	0.080	0.009	1135	3652	319	1255

Supplementary Table 3. Amplicon sequence variants (ASVs) identified at genus/group or species levels modified by any of the treatments compared to the control. **Please see Supplemental excel file: Supplementary Table 3.**

Supplementary Table 4. Information on the immune anti-mouse and cell viability markers used for flow cytometry

Marker	Clone	Reference	Manufacturer	Dilution
CD16/CD32	2.4G2	553142	BD Bioscience	1:50
CD25	PC61	557658	BD Bioscience	1:25
CD3ε	145-2C11	551163	BD Bioscience	1:50
CD4	GK1.5	563050	BD Bioscience	1:150
TCRαβ	H57-597	742485	BD Bioscience	1:50
CD115 (CSF-1R)	T38-320	567027	BD Bioscience	1:200
I-A/I-E (MHC-II)	2G9	743870	BD Bioscience	1:200
CD11c	HL3	558079	BD Bioscience	1:200
Lineage Antibody Cocktail	145-2CII; RB6-8c5; RA3-6B2; Ter-119; M1/70	561317	BD Bioscience	1:5
CD206	C068C2	141716	Biolegend	1:100
CD45.2	104	109824	Biolegend	1:200
CD127 (IL7ra)	A7R34	135024	Biolegend	1:50
Thy1.2 (CD90.2)	53-2.1	140317	Biolegend	1:200
iNOS	CXNFT	17-5920-80	eBioscience	1:50
NK1.1	PK136	25-5941-82	eBioscience	1:100
Nkp46	29A1.4	46-3351-82	eBioscience	1:100
CD11b	M1/70	47-0112-82	Invitrogen	1:200
CD163	TNKUPJ	11-1631-82	Invitrogen	1:200
LIVE/DEAD™ Fixable Aqua (405nm)	-	LTI L34957	Invitrogen	1:50
CD19	REA749	130-112-036	Miltenyi biotec	1:50
CD45	REA737	130-110-796	Miltenyi biotec	1:100
F4/80	REA126	130-102-327	Miltenyi biotec	1:50
Foxp3	REA788	130-111-678	Miltenyi biotec	1:50
Tbet	REA102	130-107-611	Miltenyi biotec	1:50
TCRγδ	REA633	130-109-750	Miltenyi biotec	1:10
CD2	RM2-5	130-102-615	Miltenyi biotec	1:10
CD5	REA421	130-106-205	Miltenyi biotec	1:50
CD80	16-10A1	130-102-372	Miltenyi biotec	1:20
IFNγ	REA638	130-109-723	Miltenyi biotec	1:50
Arg1	Met1-Lys322	PE	R&dSystems	1:10
CD3ε	145-2C11	100312	Biolegend	1:200
CD8a	53-6.7	100712	Biolegend	1:200
CD19	6D5	115512	Biolegend	1:200
Ly-76 (Ter119)	TER-119	116212	Biolegend	1:200
Cd11c	N418	117310	Biolegend	1:200
TCR β	H57-597	17-5961-83	eBioscience	1:200
TCRγδ	GL3	118116	Biolegend	1:200
Ly-6G/Ly-6C(Gr1)	RB6-8C5	108412	Biolegend	1:400
CD11b	M1/70	17-0112-83	eBioscience	1:400

Supplementary Table 5. Gene names, abbreviations and primer sequences or *TaqMan* references use for gene expression analysis.

Gene name (abbreviation)	Sequence 5´- 3´and TaqMan Gene reference
CCAAT/enhancer binding protein beta *(Cebpb)*	F: TCGGGACTTGATGCAATCC<br>R: AAACATCAACAACCCCGC
Chemokine (C-C motif) ligand 2 *(Ccl2)*	F: GCAGTTAACGCCCCACTCA<br>R: CCCAGCCTACTCATTGGGATCA
Claudin 3 *(Cldn3)*	F: TCATCGGCAGCAGCATCATCAC<br>R: ACGATGGTGATCTTGGCCTTGG
Cluster of differentiation 36 (Cd36)	F: GCCAAGCTATTGCGACATGA<br>R: ATCTCAATGTCCGAGACTTTTCAAC
Granzime B (*GrB*)	F: GCCCACAACATCAAAGAACAG<br>R: AACCAGCCACATAGCACACAT
Integrin αEβ7 (*αEβ7*)	F: CCTGTGCAGCATGTAAAAGAATG<br>R: CAAGGATCGGCAGTTCAGATAC
Integrin, alpha X *(Itgax or cd11c)*	F: ACGTCAGTACAAGGAGATGTTGGA<br>R: ATCCTATTGCAGAATGCTTCTTTACC
Interleukin 10 *(Il10)*	F: GCTCTTACTGACTGGCATGAG<br>R: CGCAGCTCTAGGAGCATGTG
Interleukin 22 *(Il22)*	F: GACATAAACAGCAGGTCCAGTT<br>R: AGAAGGCTGAAGGAGACAGT
Interleukin 6 *(Il6)*	F: ACAAGTCGGAGGCTTAATTACACAT<br>R: TTGCCATTGCACAACTCTTTC
Lipo-protein lipase *(Lpl)*	F: TGAAAGCCGGAGAGACTCAG<br>R: AGTGTCAGCCAGACTTCTTCAG
Occludin (Ocln)	F: ATGTCCGGCCGATGCTCTC<br>R: TTTGGCTGCTCTTGGGTCTGTAT
Phospholipase A2 group IIA (*Pla2g2a*)	F: AAGGATCCCCCAAGGATGCCAC<br>R: CAGCCGTTTCTGACAGTTCTGG
Regenerating islet-derived protein 3 gamma (*Reg3γ*)	F: TTCCTGTCCTCCATGATCAAA<br>R: CATCCACCTCTGTTGGGTTC
Ribosomal protein 19 (*Rpl19*)	F: CCTTGTCTGCCTTCAGCTTGT<br>R: GAAGGTCAAAGGGAATGTGTTCA
Tumor necrosis factor alpha *(Tnfa)*	F: TGTCTCAGCCTCTTCTCATTCC<br>R: TGAAAATCTGGGCCATAGAAC
Glyceraldehyde-3-phosphate dehydrogenase (Gapdh)	Mm99999915_g1
Hypoxanthine-guanine phosphoribosyltransferase (Hprt)	Mm00446968_m1
Interferon gamma *(Ifng)*	Mm01168134_m1
Interleukin 22 *(Il22)*	Mm01226722_g1
Interleukin 23 receptor (*IL23r*)	Mm00519943_m1
RAR-related orphan receptor c *(Rorgc)*	Mm01261022_m1
T-box transcription factor 21 *(Tbx21)*	Mm00450960_m1
Tumor necrosis factor alpha (*Tnfa)*	Mm00443260_g1
