# Supplementary material for: Gut commensal Phascolarctobacterium faecium retunes innate immunity to mitigate obesity and metabolic disease in mice
Source: Nat Microbiol. 2025 May 6;10(6):1310–22. doi: 10.1038/s41564-025-01989-7 (PMC12137122; doi:10.1038/s41564-025-01989-7)
Supplement: Supplementary file 2 — Reporting Summary [file 41564_2025_1989_MOESM2_ESM.pdf]

nature portfolio

Corresponding author(s): Prof. Yolanda Sanz

Last updated by author(s): 2025/03/14

Reporting Summary

Nature Portfolio wishes to improve the reproducibility of the work that we publish. This form provides structure for consistency and transparency in reporting. For further information on Nature Portfolio policies, see our Editorial Policies and the Editorial Policy Checklist.

Statistics

For all statistical analyses, confirm that the following items are present in the figure legend, table legend, main text, or Methods section.

n/a	Confirmed	
☐	☒	The exact sample size (*n*) for each experimental group/condition, given as a discrete number and unit of measurement
☐	☒	A statement on whether measurements were taken from distinct samples or whether the same sample was measured repeatedly
☐	☒	The statistical test(s) used AND whether they are one- or two-sided<br>*Only common tests should be described solely by name; describe more complex techniques in the Methods section.*
☐	☒	A description of all covariates tested
☐	☒	A description of any assumptions or corrections, such as tests of normality and adjustment for multiple comparisons
☐	☒	A full description of the statistical parameters including central tendency (e.g. means) or other basic estimates (e.g. regression coefficient) AND variation (e.g. standard deviation) or associated estimates of uncertainty (e.g. confidence intervals)
☐	☒	For null hypothesis testing, the test statistic (e.g. *F*, *t*, *r*) with confidence intervals, effect sizes, degrees of freedom and *P* value noted<br>*Give P values as exact values whenever suitable.*
☒	☐	For Bayesian analysis, information on the choice of priors and Markov chain Monte Carlo settings
☒	☐	For hierarchical and complex designs, identification of the appropriate level for tests and full reporting of outcomes
☒	☐	Estimates of effect sizes (e.g. Cohen's *d*, Pearson's *r*), indicating how they were calculated

Our web collection on statistics for biologists contains articles on many of the points above.

Software and code

Policy information about availability of computer code

Data collection	No special sofware was used for data collection
Data analysis	GraphPad software (v.9, San Diego, CA)was used for main data analysis.<br>Quantification of amplicon sequence variants (ASVs) was performed with the DADA2 v.1.24 R package<br>Alpha diversity was calculated through the estimation of the observed ASVs, Shannon index and Inverse Simpson index using the Phyloseq v.1.40 R package.<br>The analysis of the differential abundance of murine microbial taxa was performed using DESeq2 v.1.36 R package.<br>Flow cytometer data were analyzed using FCS express v.5 flow cytometry software or FACS Diva software v.7.0.<br>For analysing 16S microbiota data from mice, we used the script: https://github.com/INNOBIOME/Macrophages_Pfaecium_MicrobiotaAnalysis. The database used was: Silva versión 138 (https://zenodo.org/records/4587955)<br>For human microbiome data analysis, we used the script "https://github.com/SegataLab/inverse_var_weight/blob/main/meta_analyses.py ." to built two datasets and the script "https://github.com/waldronlab/curatedMetagenomicDataAnalyses/blob/main/python_tools/metaanalyze.py" to run logistic regression model.

For manuscripts utilizing custom algorithms or software that are central to the research but not yet described in published literature, software must be made available to editors and reviewers. We strongly encourage code deposition in a community repository (e.g. GitHub). See the Nature Portfolio guidelines for submitting code & software for further information.

Data

Policy information about availability of data

All manuscripts must include a data availability statement. This statement should provide the following information, where applicable:

- Accession codes, unique identifiers, or web links for publicly available datasets
- A description of any restrictions on data availability
- For clinical datasets or third party data, please ensure that the statement adheres to our policy

Data supporting the findings of this study are found in the paper, the extended data figures and supplementary information, or source data files. For human studies we used human metagenomes from healthy, adult individuals with metadata availablhttps://waldronlab.github.io/curatedMetagenomicData/ and through the Bioconductor package installer.
The sequencing data corresponding to the murine microbiota generated in this study have been deposited in the European Nucleotide Archive (ENA) at EMBL-EBI, under accession number PRJEB59864. The database used was: Silva versión 138 (https://zenodo.org/records/4587955)
Additional information is available for the authors upon request.

Research involving human participants, their data, or biological material

Policy information about studies with human participants or human data. See also policy information about sex, gender (identity/presentation), and sexual orientation and race, ethnicity and racism.

Reporting on sex and gender	Sex of the participant whose metagenomes data are available in Pasolli E, Schiffer L, Manghi P, et al. Accessible, curated metagenomic data through ExperimentHub. Nat Methods. 2017;14:1023–4. doi: 10.1038/nmeth.4468
Reporting on race, ethnicity, or other socially relevant groupings	N/A
Population characteristics	Human metagenomes' characteristics used for this study are detailed in "meta-analysis of human metagenomes" in Material and Methods section.
Recruitment	N/A
Ethics oversight	N/A

Note that full information on the approval of the study protocol must also be provided in the manuscript.

Field-specific reporting

Please select the one below that is the best fit for your research. If you are not sure, read the appropriate sections before making your selection.

☒ Life sciences ☐ Behavioural & social sciences ☐ Ecological, evolutionary & environmental sciences

For a reference copy of the document with all sections, see nature.com/documents/nr-reporting-summary-flat.pdf

Life sciences study design

All studies must disclose on these points even when the disclosure is negative.

Sample size	Statistical methods were not used to pre-determine sample sizes. Sizes were chosen on the basis of establised practice and similarity to those sizes those reported in previous publications (doi: 10.1080/19490976.2020.1865706; doi: 10.1080/19490976.2023.2181928) which are proved to be sufficient to obseve significant biological effects. The sample size (n) for each experiment is provided in methods section and in the figure legends for every experiment.
Data exclusions	Grubbs' test was used for outlier detection.
Replication	The results are shown as mean ± SEM and n represent the number of biological replicates shown as individual dots. In experimental analysis all samples were run at least in duplicate. All attempts of replication were consistent.
Randomization	Mice were randomly housed in groups of 4-5 animals per cage in a ventilated rack under controlled temperature (23 ± 2°C) and relative humidity (40-50%), and with a 12-hour-light/dark cycle. In all cell experiments samples were randomly distributed and both, control and treated conditions, were included in each plate to avoid biased results due to plate position.
Blinding	During both data collection and analysis, blinding was not implemented in this study. The same researchers who designed the experiments were also responsible for performing the experiments and analyzing most of the results. This made it difficult to maintain blinding, especially since the difference between the treatment group and the vehicle group was highly visible due to the turbidity caused by the bacterium treatmen. Given this noticeable distinction, blinding was not feasible.

Reporting for specific materials, systems and methods

We require information from authors about some types of materials, experimental systems and methods used in many studies. Here, indicate whether each material, system or method listed is relevant to your study. If you are not sure if a list item applies to your research, read the appropriate section before selecting a response.

Materials & experimental systems

n/a	Involved in the study
☐	☒ Antibodies
☐	☒ Eukaryotic cell lines
☒	☐ Palaeontology and archaeology
☐	☒ Animals and other organisms
☒	☐ Clinical data
☒	☐ Dual use research of concern
☒	☐ Plants

Methods

n/a	Involved in the study
☒	☐ ChIP-seq
☐	☒ Flow cytometry
☒	☐ MRI-based neuroimaging

Antibodies

Antibodies used

The information is given as " target name, clone, catalogue number and supplier name" for every antibodie. We do not have the specific lote number used in every experiment.
CD16/CD32 (clone 2.4G2) 553142 BD Bioscience;
CD25 (clone PC61) 557658 BD Bioscience;
CD3ε(clone 145-2C11) 551163 BD Bioscience;
CD4 (clone GK1.5) 563050 BD Bioscience;
TCRαβ (clone H57-597) 742485 BD Bioscience;
CD115 (clone T38-320) 567027 BD Bioscience;
I-A/I-E (MHC-II) (clone 2G9) 743870 BD Bioscience;
CD11c (clone HL3) 558079 BD Bioscience;
Lineage Antibody Cocktail (clones: 145-2CII; RB6-8c5; RA3-6B2; Ter-119; M1/70) 561317 BD Bioscience;
CD206 (clone C068C2) 141716 Biolegend;
CD45.2 (clone 104) 109824 Biolegend;
CD127 (clone A7R34) 135024 Biolegend;
Thy1.2 (CD90.2) (clone 53-2.1) 140317 Biolegend;
iNOS (clone CXNFT) 17-5920-80 eBioscience;
NK1.1 (clone PK136) 25-5941-82 eBioscience;
Nkp46 (clone 29A1.4) 46-3351-82 eBioscience;
CD11b (clone M1/70) 47-0112-82 Invitrogen;
CD163 (clone TNKUPJ) 11-1631-82 Invitrogen;
LIVE/DEAD™ Fixable Aqua (405nm) LTI L34957 Invitrogen;
CD19 (clone REA749) 130-112-036 Miltenyi biotec;
CD45 (clone REA737) 130-110-796 Miltenyi biotec;
F4/80 (clone REA126) 130-102-327 Miltenyi biotec;
Foxp3 (clone REA788) 130-111-678 Miltenyi biotec;
Tbet (clone REA102) 130-107-611 Miltenyi biotec;
TCRγδ (clone REA633) 130-109-750 Miltenyi biotec;
CD2 (clone RM2-5) 130-102-615 Miltenyi biotec ;
CD5 (clone REA421) 130-106-205 Miltenyi biotec ;
CD80 (clone 16-10A1) 130-102-372 Miltenyi biotec ;
IFNϒ (clone REA638) 130-109-723 Miltenyi biotec ;
Arg1 (clone Met1-Lys322) PE R&dSystems;
CD3ε (clone 145-2C11) 100312 Biolegend;
CD8a (clone 53-6.7) 100712 Biolegend;
CD19 (clone 6D5) 115512 Biolegend;
Ly-76 (Ter119) (clone TER-119) 116212 Biolegend;
Cd11c (clone N418) 117310 Biolegend;
TCR β (clone H57-597) 17-5961-83 eBioscience;
TCRγδ (clone GL3) 118116 Biolegend;
Ly-6G/Ly-6C(Gr1) (clone RB6-8C5) 108412 Biolegend;
CD11b (clone M1/70) 17-0112-83 eBioscience;
Anti-TLR2 (clone C9A12) #MABG-MTLR2-2, InvivoGen.

Validation

The validation of all commertial primary antibodies used for the species and application can be found on the manufacture's websites. Since all antibody lots are routinely tested by providers and each product comes with a certificate of analysis from indicated vendor stating that the product met all quality control standards, no extra validation process was done in our laboratory.

Eukaryotic cell lines

Policy information about cell lines and Sex and Gender in Research

Cell line source(s)	We used isolated murine cells (Intestinal ILC1s and bone marrow derived macrophages) and HEK-Blue hTLR2 cell lines (HEK293 cells #hkb-htlr2, Invivogen, CA, USA)
Authentication	Details of isolation and authentification procedures are exhaustively described in material and methods section.
Mycoplasma contamination	HEK-Blue hTLR2 cell were cetifyed to be free of mycoplasma contamination by manufacturer. Exvivo cell cultures were not tested for mycoplasma contamination.
Commonly misidentified lines (See ICLAC register)	Not used in the present study.

Animals and other research organisms

Policy information about studies involving animals; ARRIVE guidelines recommended for reporting animal research, and Sex and Gender in Research

Laboratory animals	Seven-week-old C57BL/6J wild type male mice and Rag1-/- male mice with a C57BL/6J background were used in the study.
Wild animals	This study did not involve wild animals.
Reporting on sex	Only male mice were used in this study in view of standard procedures. However, future studies in females are need it to identify any diferences based on sex.
Field-collected samples	No field collected sample was used in the study.
Ethics oversight	Animal procedures were evaluated and approved by the ethics committee of the University of Valencia (Animal Production Section, SCSIE, University of Valencia) and authorized by the competent authority (Generalitat Valenciana) who assigned the following approval IDs: 2017/VSC/PEA/00015, 2018/VSC/PEA/0171, 2021/VSC/PEA/0177 and 2024/VSC/PEA/0126. The procedures conformed to EU directive 2010/63/UE and the Spanish RD53/2013 regulation, regarding the protection of animals used for experimental and other scientific purposes.

Note that full information on the approval of the study protocol must also be provided in the manuscript.

Plants

Seed stocks	N/A
Novel plant genotypes	N/A
Authentication	N/A

Flow Cytometry

Plots

Confirm that:

☒ The axis labels state the marker and fluorochrome used (e.g. CD4-FITC).

☒ The axis scales are clearly visible. Include numbers along axes only for bottom left plot of group (a 'group' is an analysis of identical markers).

☒ All plots are contour plots with outliers or pseudocolor plots.

☒ A numerical value for number of cells or percentage (with statistics) is provided.

Methodology

Sample preparation	Sample preparation is exhaustively detailed in "isolation of intestinal immune cells and flow cytometry analysis" design in Material and Methods section.

Instrument	Data were acquired with a BD LSRFortessa flow cytometer and cells were sorted in Aria Cell sorter (Becton Dickinson Biosciences).
Software	FCS express v.5 flow cytometry software or FACS Diva software v.7.0 (BD Biosciences).
Cell population abundance	According to our data the abundance of sorted ILC1 was 1% of CD45+ cells and post-sorted purity is stimated to be greater than 95% according to optimized protocol doi: 10.1016/j.celrep.2015.02.057.
Gating strategy	Gating strategy was done according to standarized protocols from our group and collaborators: doi.org/10.1080/19490976.2020.1865706 and doi: 10.1038/s41586-019-1579-3. It can be found in Extended data figures 4 and 5.

☒ Tick this box to confirm that a figure exemplifying the gating strategy is provided in the Supplementary Information.
